# ALT1, a Snf2 Family Chromatin Remodeling ATPase, Negatively Regulates Alkaline Tolerance through Enhanced Defense against Oxidative Stress in Rice

**DOI:** 10.1371/journal.pone.0112515

**Published:** 2014-12-04

**Authors:** Mingxin Guo, Ruci Wang, Juan Wang, Kai Hua, Yueming Wang, Xiaoqiang Liu, Shanguo Yao

**Affiliations:** 1 State Key Laboratory of Plant Genomics and National Plant Gene Research Center, Institute of Genetics and Developmental Biology, Chinese Academy of Sciences, Beijing, China; 2 University of Chinese Academy of Sciences, Beijing, China; Key Laboratory of Horticultural Plant Biology (MOE), China

## Abstract

Alkaline salt stress adversely affects rice growth, productivity and grain quality. However, the mechanism underlying this process remains elusive. We characterized here an alkaline tolerant mutant, *alt1* in rice. Map-based cloning revealed that *alt1* harbors a mutation in a chromatin remodeling ATPase gene. *ALT1*-RNAi transgenic plants under different genetic background mimicked the *alt1* phenotype, exhibiting tolerance to alkaline stress in a transcript dosage-dependent manner. The predicted ALT1 protein belonged to the Ris1 subgroup of the Snf2 family and was localized in the nucleus, and transcription of *ALT1* was transiently suppressed after alkaline treatment. Although the absorption of several metal ions maintained well in the mutant under alkaline stress, expression level of the genes involved in metal ions homeostasis was not altered in the *alt1* mutant. Classification of differentially expressed abiotic stress related genes, as revealed by microarray analysis, found that the majority (50/78) were involved in ROS production, ROS scavenging, and DNA repair. This finding was further confirmed by that *alt1* exhibited lower levels of H_2_O_2_ under alkaline stress and tolerance to methyl viologen treatment. Taken together, these results suggest that *ALT1* negatively functions in alkaline tolerance mainly through the defense against oxidative damage, and provide a potential two-step strategy for improving the tolerance of rice plants to alkaline stress.

## Introduction

The widely distributed salt and alkaline salts are important abiotic stress factors that greatly affect plant growth and development and severely threat crop productivity throughout the world [1]. Previous research has demonstrated that alkaline salt stress and neutral salt stress are two distinct kinds of stresses for plants, and should be called alkali stress and salt stress, respectively [2]. Alkaline salts (NaHCO_3_ and Na_2_CO_3_), which elevate soil pH, are much more destructive than neutral salts (NaCl and Na_2_SO_4_). So far, extensive studies have tried to uncover the signaling mechanisms and regulatory networks underlying plant tolerance to salt stress [3]–[6]. However, few regulators have been identified to function directly in the tolerance of plants to alkaline salt stress.

Alkaline salt stress involves multiple factors including osmotic stress, ion injury, and elevated soil pH (pH>8.5), which reduces iron (Fe) solubility [7]. Consequently, plants grown in calcareous soils often exhibit Fe deficiency symptoms of chlorosis [8], [9]. Therefore, various groups have sought to genetically engineer crop plants with improved Fe uptake under alkaline salt conditions, by introducing genes encoding iron transporters, iron reductases, and enzymes involved in phytosiderophore biosynthesis into plants [8]–[14]. For example, transgenic rice expressing the barley nicotianamine aminotransferase gene, *HvNAAT1*, showed enhanced Fe availability and higher grain yields in alkaline soils [8]. Similarly, ectopic expression of the yeast Fe^3+^-chelate-reductase gene *refre1/372* greatly improved grain yield on calcareous soil-grown rice plants [9].

In addition to the strategies that overcome the Fe limitations of alkaline soils, a series of studies suggested that many other mechanisms appear to be involved in the tolerance to alkaline salt stress in plants. For example, analysis of the global gene expression profiles of *Puccinellia tenuiflora*, a widely distributed monocotyledonous alkaline-tolerant halophyte in the Songnen Plains of China, under alkaline salt stress (Na_2_CO_3_ or NaHCO_3_) revealed that the differentially expressed genes fell into a dozen functional categories, implying the complex mechanisms underlie alkaline salt tolerance in plants [15]. In *Arabidopsis*, protein kinase PKS5 was reported to negatively regulate tolerance to high external pH by interacting with chaperone J3 to inhibit plasma membrane H^+^-ATPase activity [16], [17]. In addition, microRNAs, which are ubiquitous regulators of gene expression in eukaryotic organisms, were also found to be involved in alkaline salt stress response [18], [19]. On the other hand, efforts have also been put to screen for natural plant germplasms with varied alkaline tolerance, and several associated quantitative trait loci (QTLs) have been roughly mapped in rice and soybean [20]–[23]. However, none of these genes have been cloned to date.

ATP-dependent chromatin remodeling complexes affect chromatin dynamics using the energy released by ATP hydrolysis to alter histone-DNA contacts, thereby making genomic regions more accessible to the transcriptional machinery or transcription factors [24]. Therefore, chromatin remodeling plays a central role in establishing specific gene expression patterns and maintaining transcriptional states in eukaryotes. Emerging evidence shows that the chromatin remodeling ATPases of Snf2 family not only play various roles in the regulation of plant development [25], but also function in different abiotic stress responses [26]. In *Arabidopsis*, knockout *atchr12* plants shows tolerance to drought, salt and heat stresses [27], and AtBRM was reported to regulate drought tolerance [28]. The rice genome contains totally 40 Snf2 family proteins [29]. However, only one member, OsCHR4, was reported to function in early chloroplast development in adaxial mesophyll cells [30]. So far, no ATP-dependent chromatin remodeling enzymes have been reported to function in alkaline salt stress response.

Here we report the functional characterization of an alkaline tolerant mutant, *alt1*, in rice. We showed that *alt1* contains a mutation in a Snf2 family chromatin remodeling ATPase gene that functions negatively in alkaline tolerance in rice. We found that the improved tolerance of the mutant to alkaline stress was largely due to the enhanced defense against oxidative damage. Our results suggest a potential two-step strategy for improving the tolerance of rice plants to alkaline salt stress.

## Results

### Phenotypic analysis of the alt1 mutant

**Figure 1:**
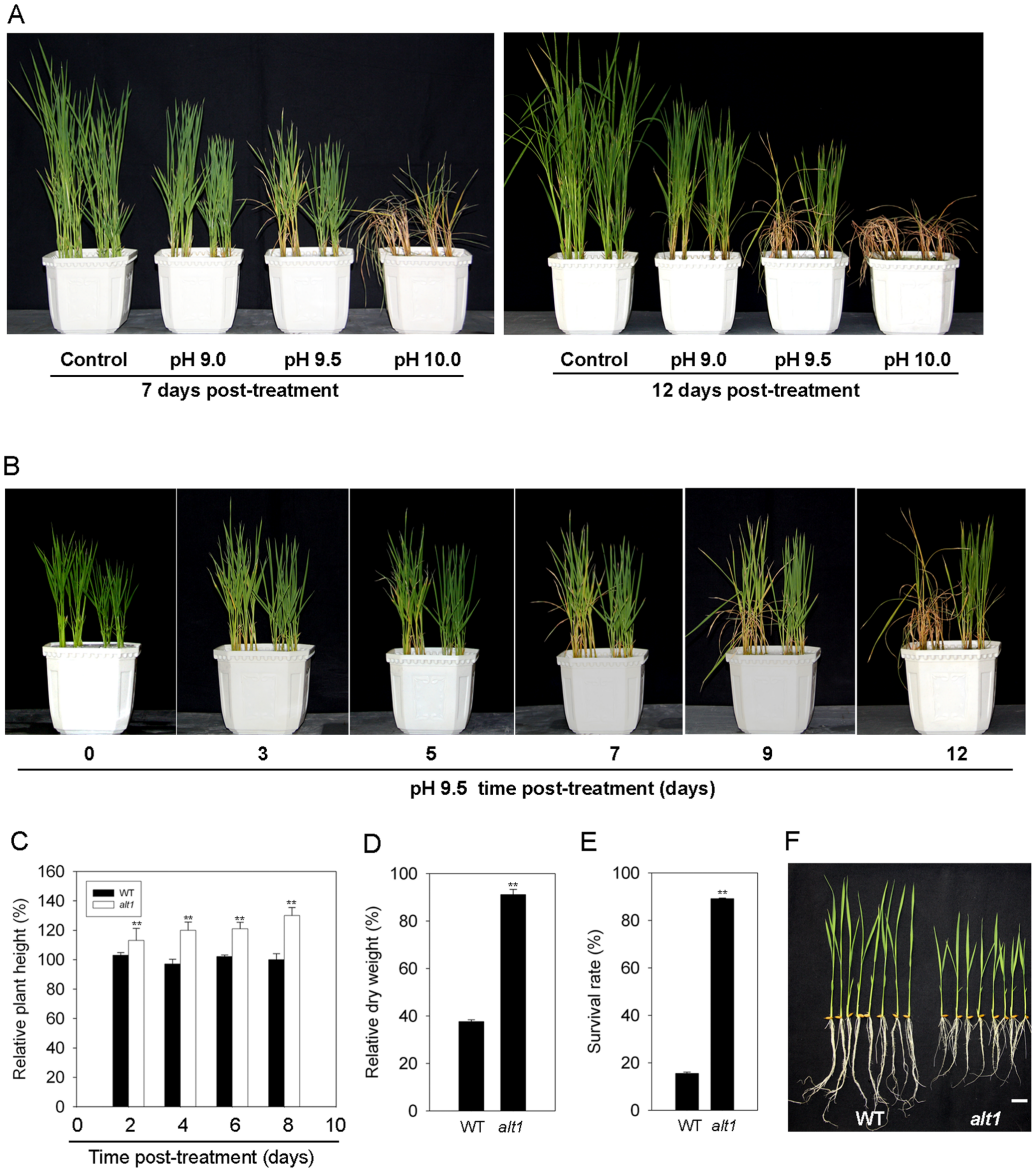
Phenotypic analysis of the *alt1* mutant. Left part in each pot of (A) and (B): WT; Right part: *alt1*. (A) Two-leaf stage *alt1* and WT seedlings were subjected to alkaline treatment with values 9.0, 9.5 and 10.0, respectively, and photographed at 7 (left) and 12 (right) days after treatment. (B) Time-course observation for tolerance phenotype of *alt1* and WT at pH 9.5. (C) Comparison of relative plant height of *alt1* and WT grown under pH 9.5 for the indicated number of days. Values are means ± SE (n = 20). **: *P*≤0.01. (D) Comparison of relative dry weight of *alt1* and WT after 10 days of treatment at pH 9.5. Values are means ± SE (n = 3, with 15 plants in each repeat). **: *P*≤0.01. (E) Comparison of survival rate of *alt1* and WT after 12 days of treatment at pH 9.5. Values are means ± SE (n = 3, with 50 plants in each repeat). **: *P*≤0.01. (F) Phenotypes of two-leaf stage seedlings of *alt1* and WT. Bar = 2 cm.

To gain insight into the molecular basis of tolerance to alkaline salt stress in rice, we screened a NaN_3_-mutagenized mutant population in the background of KY131, a widely cultivated variety in north China. From a total of 100,000 M_2_ individuals treated with NaHCO_3_-NaOH solution (pH 9.5), we identified a mutant, *alt1* (for *alkaline tolerance 1*) with enhanced tolerance to alkaline stress. To obtain detailed information on the alkaline tolerance phenotype of the mutant, two-leaf stage *alt1* and wild type (WT) plants were subjected to treatment with alkaline solutions ranging in pH from 9.0 to 10.0. The *alt1* mutant showed less chlorosis than WT under all pH values (Figure 1A). At pH 9.0, plants of *alt1* and WT grew similarly for the first 12 days of treatment (Figure 1A), but the mutant displayed an obvious tolerant phenotype 15 days after the stress (Figure S1). For pH 10.0, most of the *alt1* seedlings survived the first 7 days, but plants of both *alt1* and WT wilted completely by 12 days after treatment (Figure 1A).

The most obvious difference in tolerance phenotype between *alt1* and WT was observed for treatment at pH 9.5. The leaves of WT seedlings became chlorotic 3 days after the start of treatment, and wilted completely by 12 days (Figure 1B). In contrast, plants of *alt1* exhibited much less chlorosis and grew well compared to WT under the same stress conditions (Figure 1B). Statistical analysis of the relative plant height (height of the treated plant/height of the plant before treatment), survival rate and relative dry weight (dry weight of the treated plant/dry weight of the plant before treatment) found that the relative plant height of *alt1* increased by up to 30% under 8 days after treatment at pH 9.5, but the growth of WT plants was stunted (Figure 1C). In addition, the relative dry weight of the mutant was 2.4-fold greater than that of WT after 10 days of the treatment (Figure 1D). The survival rate of *alt1* was 89% after 12 days of treatment, but only 15% for WT (Figure 1E). These data demonstrated that *alt1* is an alkaline stress tolerant mutant.

To investigate whether the *ALT1* mutation also affected other abiotic stress processes, we next examined the phenotype of *alt1* and WT plants under salt or drought stress. No differences were found between the mutant and WT under NaCl or dehydration treatment (Figure S2). These observations suggested that tolerance to salt or drought stress was not enhanced in the *alt1* mutant.

The most evident morphological alteration of the *alt1* mutant is short roots during early development (Figure 1F). At the adult stage, no significant morphological alterations were found between *alt1* and WT plants, with the exception of tiller number per plant (Table S1).

### Map-based cloning and complementation test of alt1

**Figure 2:**
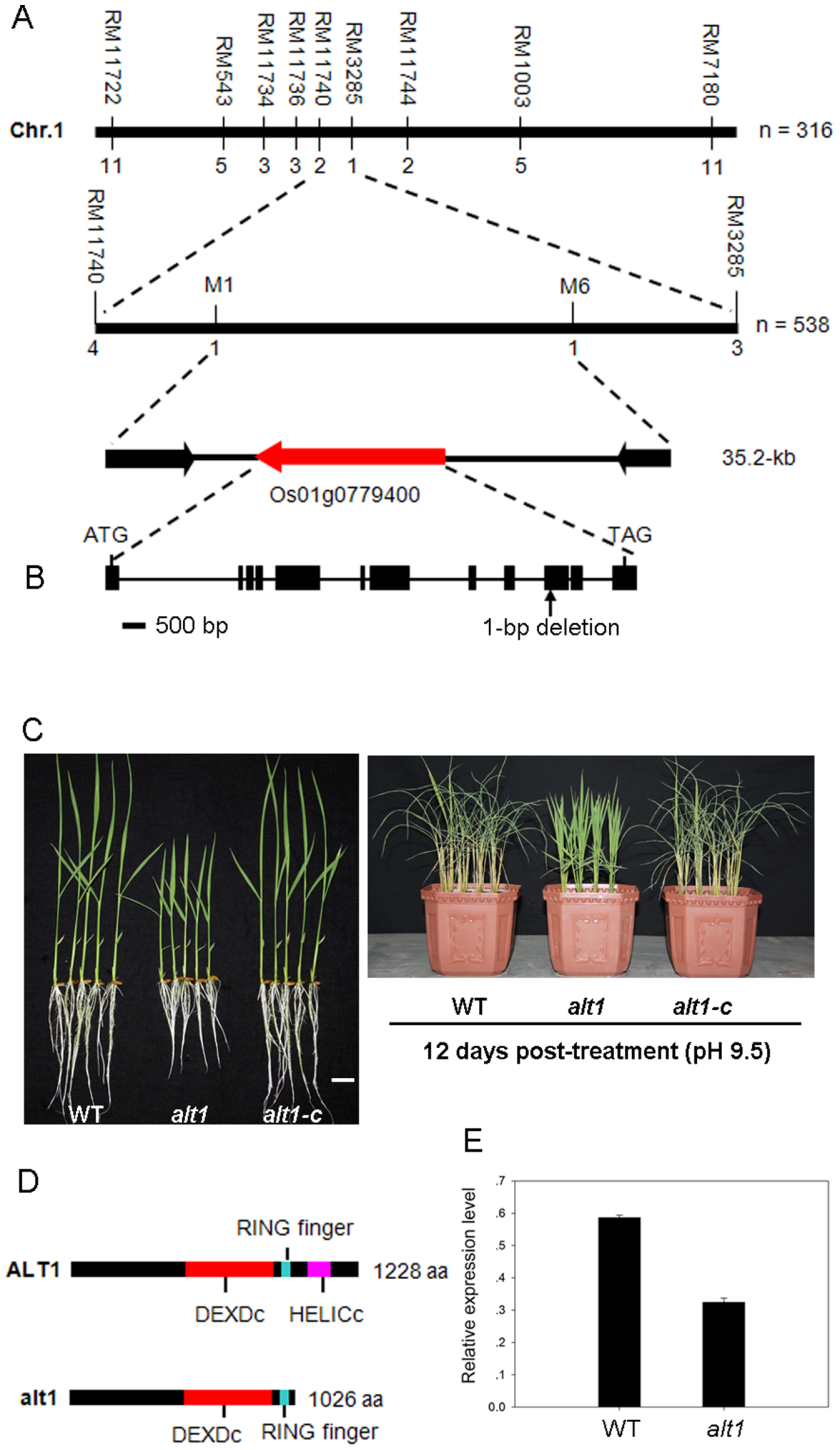
Map-based cloning of *ALT1* and complementation analysis. (A) Fine mapping of the *ALT1* locus. Numbers below the horizontal line are the number of recombinants. The *ALT1* locus was fine mapped to a 35.2-kb region between markers M1 and M6. Indicating three putative ORFs contained in this region. (B) Gene structure of *ALT1*. Black boxes indicate exons and lines between boxes indicate introns. There is 1-bp deletion in the 10^th^ exon in the *alt1* background. (C) Phenotypic analysis of two-leaf stage seedlings of WT, *alt1* and *alt1*-c under normal (left) and alkaline (right, pH 9.5) stress conditions, respectively. Bar = 2 cm. (D) Protein structure of ALT1 and alt1. (E) Transcript levels of *ALT1* in the roots of two-leaf-stage *alt1* and WT seedlings. *Actin* was used as an internal control. Data shown are mean values of three biological repeats with SD.

As described above, the *alt1* mutant showed enhanced alkaline tolerance as well as phenotypes of short roots and low tiller number. Because individuals with the mutant short root phenotype could be easily distinguished among the segregating population, we used this trait for mapping analysis. Investigation of the short root phenotype in the F_2_ population derived from a cross between *alt1* and WT revealed that *alt1* is caused by a single recessive nuclear gene mutation (χ^2^ = 0.28<χ^2^_0.05_ = 3.84; *P*>0.05). Therefore, we performed map-based cloning to isolate the underlying gene. Using 538 F_2_ mutant plants derived from the cross between *alt1* and Kasalath, the candidate gene was narrowed down to a 35.2-kb region between STS markers M1 and M6 on the long arm of chromosome 1 (Figure 2A). Three predicted genes exist within this region. After sequencing these genes, only a single nucleotide deletion (G) was identified at the 3037^th^ position of *Os01g0779400* in *alt1* (Figure 2B).

To verify that *Os01g0779400* is indeed *ALT1*, we performed a genetic complementation test in which *alt1* plants were transformed with wild-type *Os01g0779400*, which consists of the full-length coding sequence of the gene driven by its native promoter (1.1-kb genomic DNA fragment upstream of the ATG start codon). All the 23 independent T_2_ transgenic lines showed root length comparable to that of control, and displayed alkaline stress (pH 9.5) response similar to that of the WT (Figure 2C). This result indicates that the mutation in *Os01g0779400* is responsible for the phenotype of short root and alkaline tolerance exhibited in the *alt1* mutant.

*ALT1* is predicted to encode a core subunit of the Snf2 family chromatin remodeling ATPase, containing the featured Snf2 ATPase domains of DEXDc and HELICc (Figure 2D) that together have at least 12 characteristic sequence motifs with various roles in nucleic acid and/or nucleotide binding or hydrolysis [31]. The rice genome contains two paralogs of *ALT1* (i.e., *Os04g0629300* and *Os08g0180300*), with identities of 53% and 56% at the protein level, respectively. All these proteins belong to the Ris1 subgroup of the Snf2 family [29]. The nucleotide deletion in the mutant not only moderately affected *ALT1* transcription (Figure 2E), but also created a premature stop codon in the predicted coding region (Figure S3), resulting in a truncated alt1 protein lacking the HELICc domain (Figure 2D).

### dsRNAi knockdown transgenic plants mimic the alt1 phenotype

**Figure 3:**
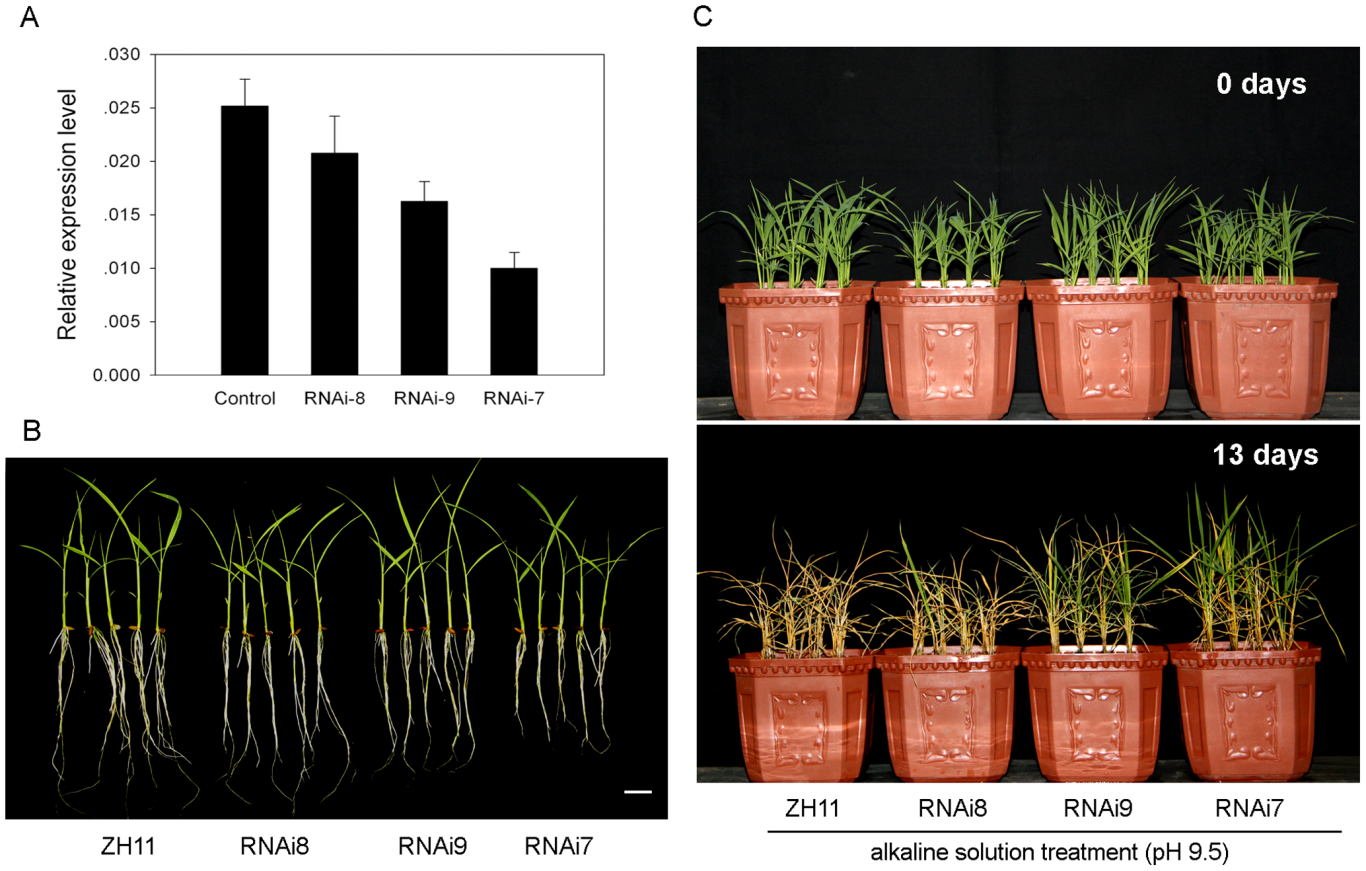
Suppression of *ALT1* resulted in enhanced tolerance to alkaline stress. (A) Transcript levels of *ALT1* in the three selected RNAi transgenic lines and the vector control. *Actin* was used as an internal control. Data shown are mean values of three biological repeats with SD. (B) Morphology of two-leaf stage seedlings of the three RNAi transgenic lines and the control cultured in tap water under natural conditions. Bar = 2 cm. (C) Phenotypic analysis of alkaline tolerance. Two-leaf stage seedlings of the three RNAi transgenic lines and the vector control were treated with alkaline solution (pH 9.5), and photographed at 0 day and 13 day after the start of treatment, respectively.

To further confirm the function of *ALT1* in alkaline tolerance, we generated a double stranded RNA interference (dsRNAi) construct harboring a unique fragment from 3516 bp to 3882 bp downstream of the start code under ZH11 background. Among the 27 independent transgenic lines generated, three lines, designated as RNAi-8, RNAi-9, and RNAi-7, were selected for their variously down-regulated *ALT1* transcription (20%, 40%, and 60%, in turn) (Figure 3A). The root length of the RNAi transgenic lines decreased in a transcript dosage-dependent manner (Figure 3B), with about 98%, 85%, and 70% of that of the WT roots. Alkaline stress (pH 9.5) treatment showed that the three RNAi transgenic plants displayed different degrees of alkaline tolerance compared with WT (Figure 3C). RNAi-7, which had the greatest reduction in transcript, showed the strongest tolerance to alkaline stress. In contrast, RNAi-8, which had the smallest reduction in transcript, displayed similar phenotype as the WT in terms of tolerance to alkaline stress (Figure 3C). After 13 days of alkaline treatment, the WT seedlings were completely wilted (Figure 3C), while the seedlings of the RNAi-7 and RNAi-9 transgenic lines remained a survival rate of 88% and 56%, respectively, under the same stress conditions. These data clearly demonstrated that *ALT1* plays a role in regulating tolerance to alkaline stress.

### Subcellular localization and transcriptional response of ALT1 to alkaline stress

**Figure 4:**
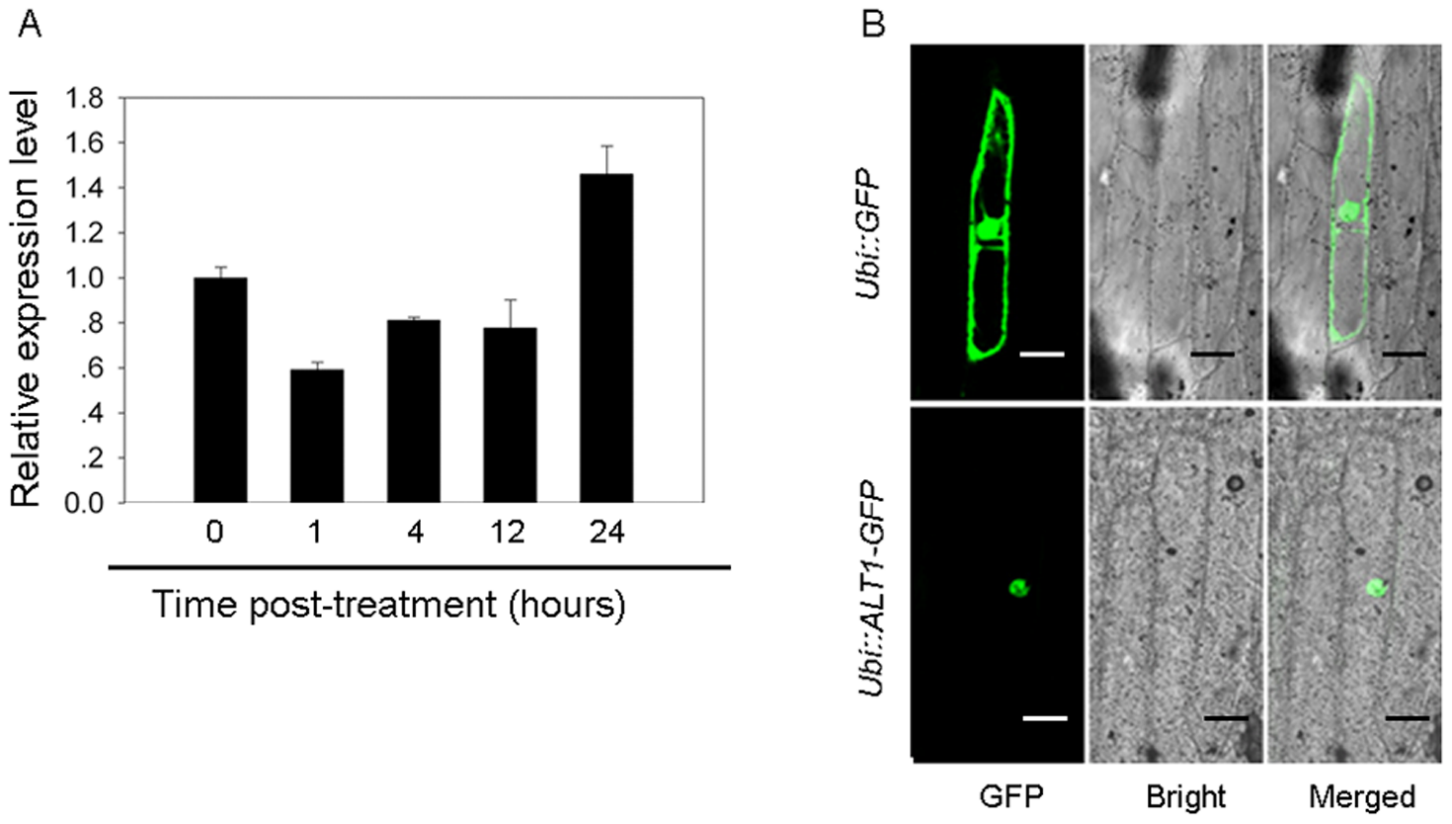
Expression and subcellular localization of ALT1. (A) Transcriptional response of *ALT1* to alkaline stress. Two-leaf stage WT seedlings were treated with alkaline solution (pH 10.0), and *ALT1* expression was monitored at the indicated time points by qRT-PCR analysis. *Actin* was used as internal control. Data shown are mean values of three biological repeats with SD. (B) Subcellular localization of ALT1. GFP and the ALT1-GFP fusion under the control of the maize *Ubi* promoter were transiently expressed in onion epidermal cells. Indicating the ALT1-GFP fusion protein was specifically expressed in the nucleus. Bars = 100 µm.

The data above showed that mutation of *ALT1* enhances tolerance to alkaline stress. For functional characterization of *ALT1*, we investigated the transcriptional response of *ALT1* to alkaline stress. The result showed that transcription of *ALT1* was transiently and moderately suppressed after 1 hour of pH 10.0 treatment (Figure 4A), consistent with the negative function of *ALT1* in alkaline tolerance.

To determine the subcellular localization of ALT1, we fused in-frame of *ALT1*, driven by the maize *ubiquitin* promoter, to the upstream of the GFP-coding sequence, and transiently expressed the construct in onion epidermal cells. The green fluorescence signal resulting from ALT1::GFP was detected exclusively in the nucleus (Figure 4B), indicating that ALT1 is a nucleus localized protein.

### alt1 shows stable capacity for ion absorption under alkaline stress

**Figure 5:**
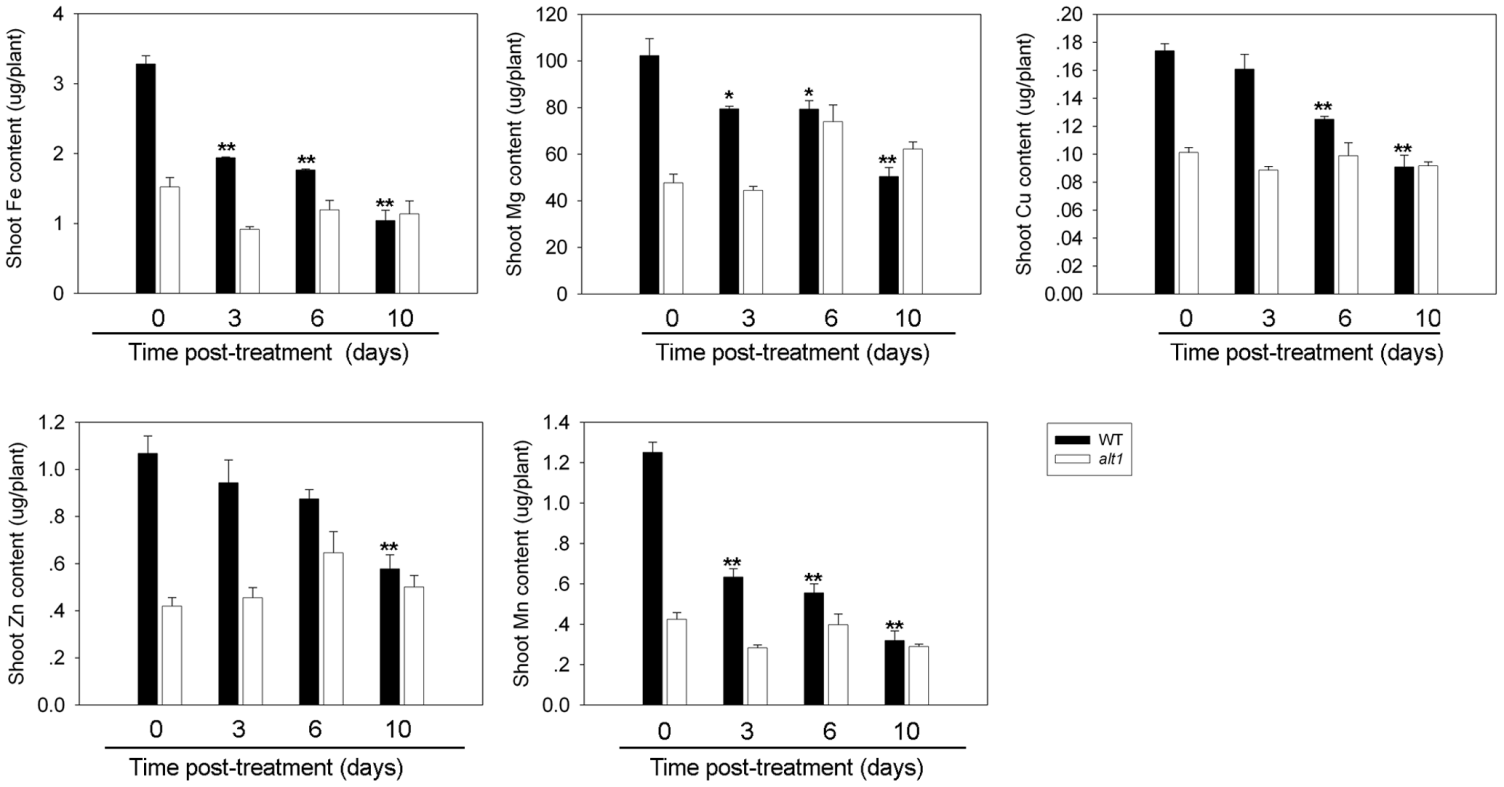
Metal ion quantification. Two-leaf stage *alt1* and WT seedlings grown hydroponically were subjected to alkaline solution (pH 9.5), and quantification of metal ions was carried out in the shoots of *alt1* and WT plants on days 0, 3, 6 and 10, respectively. Values are means ± SE (n = 3). Asterisks denote significance compared with the control plants of day 0. *: *P*≤0.05, **: *P*≤0.01.

As alkaline stress severely affects the absorption of metal ions especially Fe [8], [9], [32], we quantified a series of metal ions (i.e., Fe, Mg, Cu, Zn, and Mn) in the shoots of *alt1* and WT under alkaline stress (pH 9.5). The quantity of all metal ions decreased significantly in WT plants after alkaline treatment (Figure 5). After 10 days of treatment, the quantity of Fe, Mg, Cu, Zn, and Mn in WT plants decreased to 30%, 50%, 53%, 53% and 23% of the starting concentrations, respectively (Figure 5). The plants of *alt1* contained lower levels of the five metal ions than the WT at the start of treatment, which should be due to the retarded growth of the mutant seedlings (Figure 1F). However, all these metal ions maintained at stable levels throughout the alkaline treatment in the mutant plants (Figure 5). These data suggest that the absorption of these metal ions, including Fe, was less affected in *alt1* under alkaline conditions.

### alt1 maintains intact root morphology under alkaline stress

**Figure 6:**
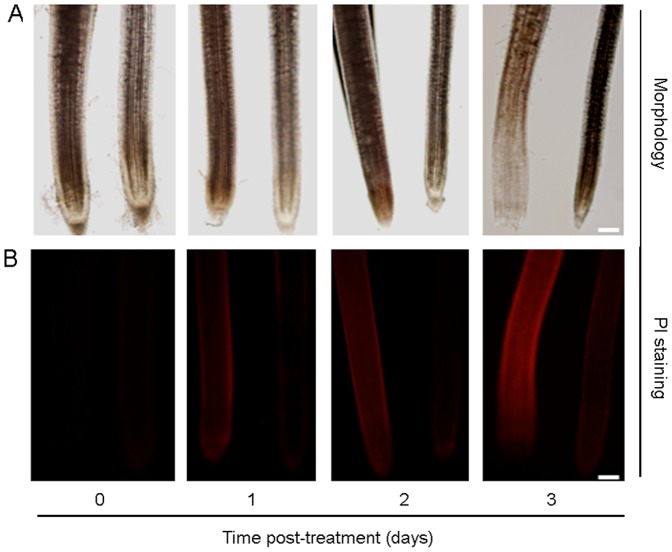
*alt1* showed intact root morphology under alkaline stress. Two-leaf stage *alt1* and WT seedlings were subjected to alkaline solution (pH 9.0) and evaluated over a time course. Left: WT; Right: *alt1*. (A) Root morphology of *alt1* and WT under alkaline treatment. (B) Propidium iodide staining of root cells of *alt1* and WT plants subjected to alkaline treatment. Bars = 2 mm.

Previous studies showed that abiotic stresses such as drought and salt usually induce plant root death [33], [34]. Our data presented above show that *alt1* had better growth (Figure 1C and 1D), and the mutant displayed a stable capacity for metal ion utilization under alkaline treatment (Figure 5). To determine whether root growth of *alt1* was differentially impaired under alkaline stress, we first examined the root morphology of plants subjected to alkaline treatment. Because roots of WT collapsed rapidly upon treatment at pH 9.5 (data not shown), we treated the seedlings with pH 9.0 for detailed comparison. The results showed that roots of *alt1* remained intact during a 3-day alkaline treatment (Figure 6A). In contrast, WT roots had obvious morphological defects after as short as 1 day of alkaline stress. Furthermore, root cap abscission was observed for WT plants after 2 days, and cells in the root tip collapsed after 3 days of treatment (Figure 6A). We next examined root vitality using PI staining, which labels dead or dying plant cells (Duan et al., 2010). Staining was detected on the root epidermis of WT after 1 day of treatment, and the stained area rapidly enlarged and intensified after the second day of treatment (Figure 6B). However, staining was consistently weak in the roots of the *alt1* mutant (Figure 6B). These observations suggest that the mutant maintained intact root morphology under alkaline stress, which might explain why roots of *alt1* are better able to absorb metal ions under such conditions.

### Oxidative stress related genes are differentially expressed in the alt1 mutant

To explore the pathway for *ALT1* to function in alkaline tolerance, a cDNA microarray analysis was performed to identify genes that are differentially expressed in *alt1* and WT. Using the Rice Gene Expression 4×44 K Microarray (Agilent Technology), we compared the gene expression profiles in roots of *alt1* and WT seedlings at the two-leaf stage under normal growth conditions. Totally, 641 transcripts were detected with differential expression levels (3-fold cutoff, P<0.01) (Table S2). Of these transcripts, 385 were up-regulated and 256 were down-regulated in *alt1* compared with WT (Table S2). Because the mutant displayed stable levels of the five metal ions including Fe under alkaline treatment (Figure 5), and the improvement of Fe utilization has been regarded as the key factor for rice plants tolerant to alkaline stress [8], [9], we first supposed that *ALT1* functions in alkaline tolerance by regulating the expression of metal ion utilization related genes. However, a survey of the microarray data found that, the known regulators functioning directly in metal ion utilization, such as *OsIDEF1* (*Os08g0101000*), *OsIDEF2* (*Os05g0426200*), *OsIRO2* (*Os01g0952800*), *OsNAS1* (*Os03g0307300*), *OsNAS2* (*Os03g0307200*), *OsNAS3* (*Os07g0689600*), *OsNAAT1* (*Os02g0306401*), *OsDMAS1* (*Os03g0237100*), *OsTOM1* (*Os12g0132650*), *OsYSL2* (*Os02g0649900*), *OsYSL15* (*Os02g0650300*), *OsIRT1* (*Os03g0667500*), and *OsIRT2* (*Os03g0667300*) [32], could not be identified among the differentially expressed genes (Table S2).

**Figure 7:**
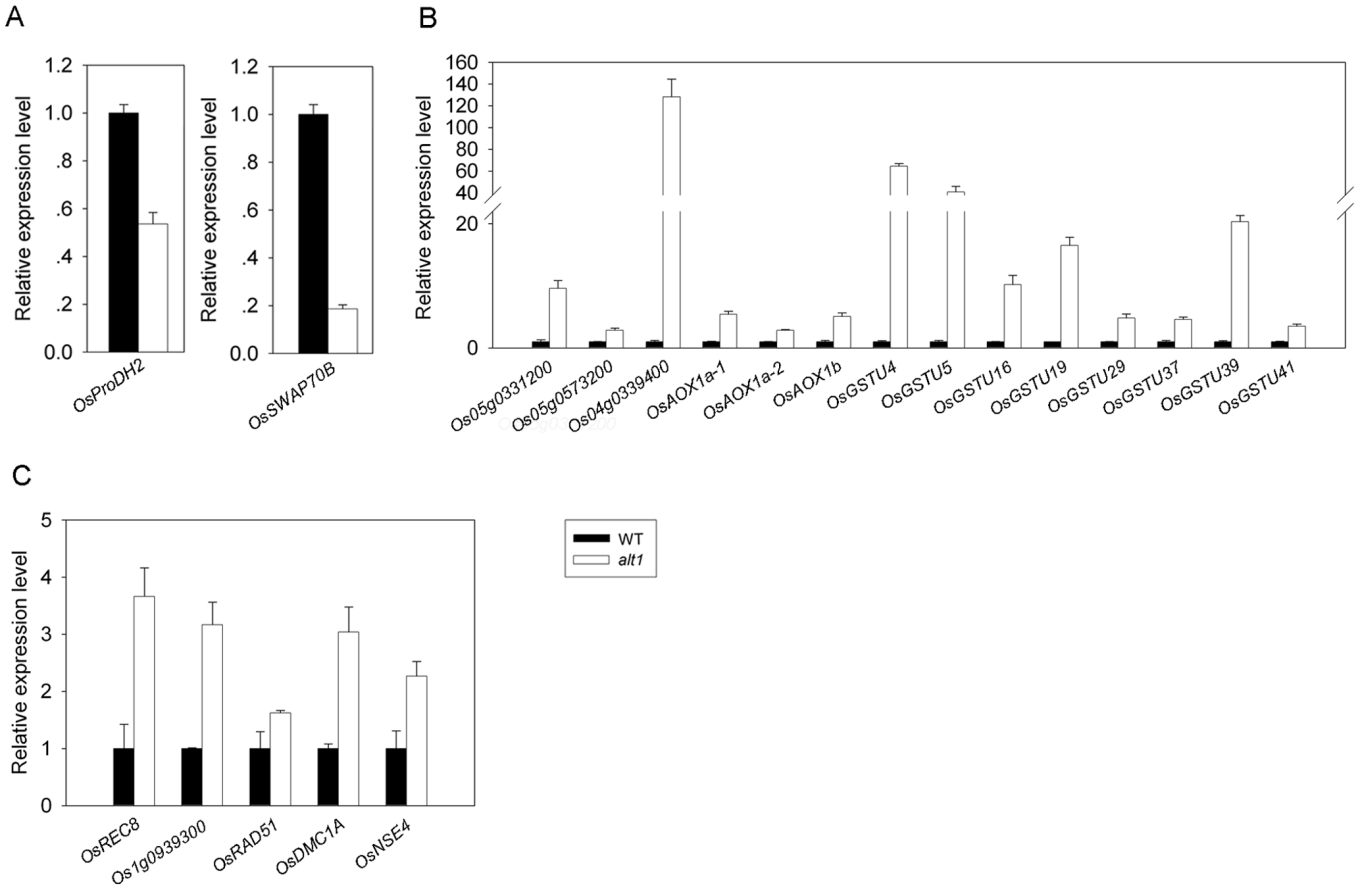
Expression analysis of oxidative stress-related genes. qRT-PCR was conducted on the roots of hydroponically grown two-leaf stage *alt1* and WT seedlings. *Actin* was used as an internal control. Data shown are mean values of three biological repeats with SD. (A) Genes related to ROS producing.- (B) Genes related to ROS scavenging. (C) Genes related to DNA repair.

Functional classification of the differentially expressed genes revealed that about 78 of them are involved in abiotic stresses (Table S2). More importantly, 50 of the 78 genes appear to be involved in the defense against oxidative stress (Table 1). For example, *OsSWAP70B* (*Os07g0138100*) functions as a suppressor of ROS production [35], and *OsProDH2* (*Os10g0550900*) appears to encode a proline oxidase that promotes ROS accumulation [36]. Transcription of both genes was significantly down-regulated in *alt1* (Figure 7A). In addition, dozens of genes appear to protect plant cells from oxidative damage by scavenging ROS, such as a batch of Glutathione *S*-transferase genes, genes involved in the mitochondrial electron transport chain, genes encoding alcohol dehydrogenase-like proteins, an isoflavone reductase-like protein, and a cysteine proteinase-like protein [37]–[42]. Most of these genes were significantly up-regulated in the mutant (Figure 7B and Table 1). These data suggest that the quantity of ROS in *alt1* might be maintained at lower levels by suppressing ROS production and enhancing ROS scavenging.

**Table 1:** Oxidative stress-related genes differentially expressed in *alt1* as revealed by microarray analysis.

Probe name^a^	Description^b^	P-value^c^	Fold change^d^
**ROS producing**			
*Os07g0138100*	Pleckstrin homology-type domain containing protein *(OsSWAP70B)*	0.000420	−5.29
*Os10g0550900*	Proline oxidase domain containing protein *(OsProDH2)*	0.000020	−6.01
**ROS scavenging**			
*Os10g0527400*	Similar to Tau class GST protein 3 *(OsGSTU19)*	0.000101	58.60
*Os01g0692100*	Glutathione S-transferase *(OsGSTU39)*	0.000003	46.22
*Os10g0525600*	Similar to Tau class GST protein 3	0.000019	42.78
*Os10g0528300*	Tau class GST protein 4 *(OsGSTU4)*	0.000161	36.84
*Os01g0949900*	Similar to Glutathione S-transferase *(OsGSTU37)*	0.000007	35.86
*Os09g0367700*	Similar to GST6 protein *(OsGSTU5)*	0.000059	25.58
*Os10g0368100*	Similar to Glutathione S-transferase GSTU35	0.000444	23.42
*Os01g0692000*	Similar to Glutathione S-transferase GST 26	0.000001	22.47
*Os05g0412800*	Similar to Glutathione S-transferase GST 41 *(OsGSTU16)*	0.000014	20.62
*Os10g0528400*	Similar to Glutathione S-transferase *(OsGSTU29)*	0.000071	11.62
*Os01g0950000*	Similar to Glutathione S-transferase GST 28 *(OsGSTU41)*	0.000352	11.56
*Os03g0595600*	Similar to Glutathione S-transferase	0.000001	9.45
*Os01g0949800*	Similar to Glutathione S-transferase GST 28	0.000001	6.75
*Os10g0530900*	Similar to Glutathione S-transferase GST 30	0.004421	6.11
*Os10g0528200*	Similar to Glutathione S-transferase TSI-1	0.000126	4.63
*Os01g0371200*	Similar to Glutathione-S-transferase 19E50	0.001183	3.87
*Os10g0525800*	Similar to Glutathione S-transferase GSTU31	0.008134	3.25
*Os10g0529500*	Similar to Glutathione-S-transferase 2	0.000013	3.23
*Os10g0529400*	Tau class GST protein 4	0.002258	3.23
*Os01g0369700*	Similar to Glutathione S-transferase GST 8 *(OsGSTF5)*	0.001745	3.00
*Os05g0148900*	Similar to Glutathione-S-transferase 19E50	0.001472	−3.29
*Os03g0643700*	Similar to GST6 protein	0.002487	−8.45
*Os02g0318100*	Alternative oxidase 1a *(OsAOX1a-1)*	0.000669	23.84
*Os04g0600200*	Alternative oxidase 1a *(OsAOX1a-2)*	0.000511	7.53
*Os05g0331200*	External rotenone-insensitive NADPH dehydrogenase	0.000550	17.33
*Os08g0141400*	Similar to External rotenone-insensitive NADPH dehydrogenase	0.000892	5.39
*Os01g0633500*	Similar to NADPH-dependent reductase A1	0.002659	−4.34
*Os02g0794600*	Similar to Copper chaperone COX17-1	0.000085	7.34
*Os09g0370200*	Copper chaperone SCO1/SenC domain containing protein	0.000104	4.78
*Os08g0496000*	Cytochrome oxidase assembly family protein	0.002097	3.12
*Os02g0791400*	Cytochrome c oxidase, subunit VIb domain containing protein	0.008472	−3.26
*Os05g0573200*	NADP-isocitrate dehydrogenase	0.001074	5.54
*Os04g0339400*	Aldo/keto reductase family protein	0.000002	236.55
*Os12g0226900*	Similar to Allyl alcohol dehydrogenase	0.000191	3.49
*Os11g0210300*	Alcohol dehydrogenase 1	0.000040	3.22
*Os02g0585700*	Quinonprotein alcohol dehydrogenase-like domain containing protein	0.001116	−5.40
*Os02g0586000*	Quinonprotein alcohol dehydrogenase-like domain containing protein	0.000832	−21.53
*Os01g0106400*	Similar to Isoflavone reductase homolog IRL *(OsIRL)*	0.000257	14.03
*Os09g0381400*	Similar to Ervatamin C	0.000013	4.33
**DNA repair**			
*Os12g0497300*	Similar to DNA repair protein RAD51 homolog *(OsRAD51)*	0.000015	8.34
*Os12g0143800*	Similar to Disrupted meiotic cDNA 1 protein *(OsDMC1A)*	0.000016	8.18
*Os11g0146800*	Similar to Disrupted meiotic cDNA 1 protein *(OsDMC1B)*	0.001043	4.79
*Os05g0580500*	Rad21/Rec8 like protein, N-terminal domain containing protein *(OsREC8)*	0.000034	7.21
*Os06g0618000*	Nse4 domain containing protein *(OsNSE4)*	0.000007	7.02
*Os01g0939300*	BRCT domain containing protein	0.000063	5.44
*Os07g0209500*	DNA-directed DNA polymerase, family B domain containing protein *(OsREV3)*	0.001388	5.03
*Os01g0801100*	Apurinic endonuclease-redox protein *(OsARP)*	0.000007	4.50
*Os05g0498300*	DNA mismatch repair protein MutS, core domain containing protein *(OsMSH5)*	0.000005	3.71

a Name of probe set on Affymetrix Rice GeneChip.

b Gene annotation in The Rice Annotation Project Database.

c P-value of statistical Student's *t*-test.

d Fold change of *alt1* compared with WT. Values are calculated by R-software.

Besides the genes involved in ROS homeostasis, a number of differentially expressed genes related to DNA repair were also identified in *alt1*, such as *OsREC8* (*Os05g0580500*) [43], *OsRAD51* (*Os12g0497300*) [44], *OsARP* (*Os01g0801100*) [45], *OsMSH5* (*Os05g0498300*) [46], *OsREV3* (*Os07g0209500*) [47], and *OsDMC1A* (*Os12g0143800*) [48]. Transcript levels of all these genes were significantly increased in the *alt1* mutant (Figure 7C and Table 1), suggesting that DNA repair machinery might be highly activated in the mutant.

We also noticed that the expression of a dozen transcription factor (TF) genes related to abiotic stresses, such as NAC, WRKY, MYB, bZIP, and C2H2-type zinc finger [49], were significantly changed in the *alt1* mutant (Table S2 and Figure S4). This result suggests that the TFs may also contribute to *ALT1*-mediated alkaline tolerance in rice.

### alt1 exhibits tolerance to oxidative stress

**Figure 8:**
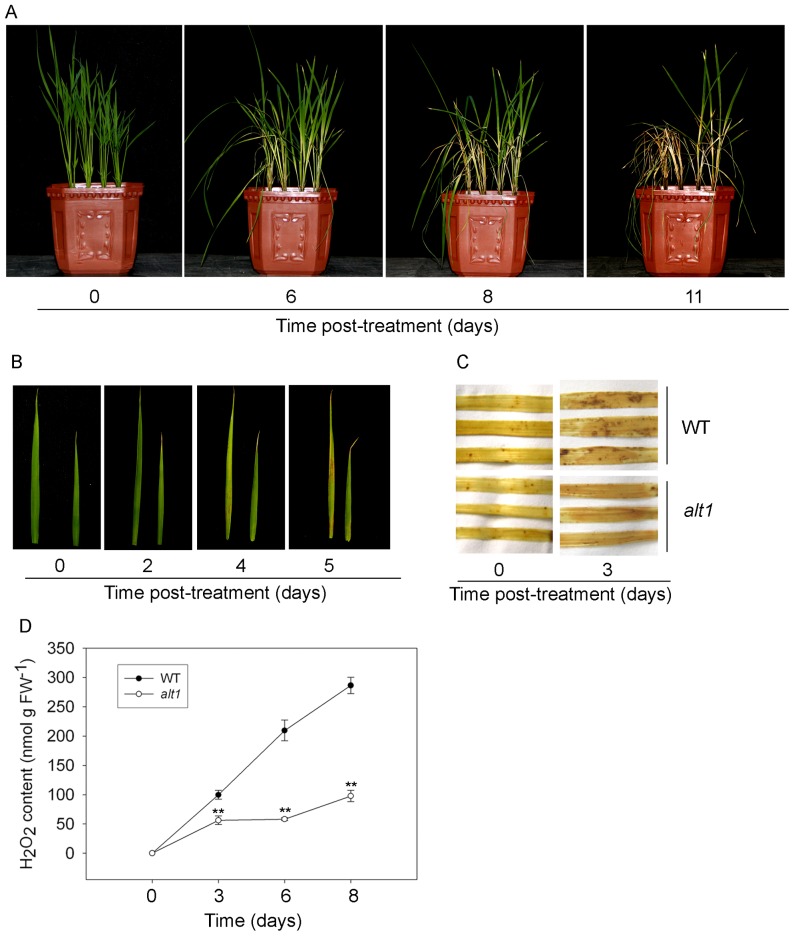
Phenotypic analysis of *alt1* and WT plants under oxidative stress. Two-leaf stage *alt1* and WT seedlings grown hydroponically were subjected to 20 µM MV treatment. The left is WT and right is *alt1* for (A) and (B), respectively. (A) Phenotypes of *alt1* and WT at the indicated time points during MV treatment. (B) Leaf morphology of *alt1* and WT during MV treatment. (C) DAB staining of *alt1* and WT leaves from plants under normal (left) and stressed (right, pH 9.5) conditions, respectively. (D) Quantitative measurement of H_2_O_2_ in *alt1* and WT leaves during pH 9.5 treatment. Values are means ± SD (n = 3).

Abiotic stresses usually cause ROS accumulation, and excessive ROS results in oxidative stress, which ultimately causes cell death [50]. The microarray data above demonstrated that expression of a majority of genes involved in oxidative stress is favorably altered in the *alt1* background, suggesting that the mutant might be tolerant to oxidative stress. To test this notion, we subjected *alt1* and WT seedlings to 20 µM methyl viologen (MV), a well-known oxidative stress inducer for the production of ROS in chloroplasts under light [51]. Four days after MV treatment, necrotic spots were visible on the leaves of WT seedlings, and WT plants began wilting after 6 days and mostly died after 11 days of MV treatment (Figure 8A). In contrast, the leaves of *alt1* were still green after 4 days of MV treatment (Figure 8B), and about half of the *alt1* plants still survived 11 days under this conditions (Figure 8A). 3, 3′-diaminobenzidine (DAB) staining showed that brown precipitate, which is indicative of H_2_O_2_ accumulation, was distributed in the leaves of both *alt1* and WT plants under MV treatment, but more precipitate was present in WT leaves than in those of *alt1* (Figure 8C). We further examined whether the alkaline stress causes higher levels of oxidative stress in WT than *alt1* by quantifying the level of H_2_O_2_. We found that the H_2_O_2_ content in WT leaves increased significantly from 0 to 300 nmol g FW^−1^ during the alkaline of pH 9.5 treatment (Figure 8D). In the leaves of the mutant, however, the level of H_2_O_2_ only reached 100 nmol g FW^−1^, one-third of that in WT after 8 days of treatment (Figure 8D). These data suggest that the oxidative stress induced by alkaline treatment is greatly alleviated in the *alt1* mutant.

## Discussion

### ALT1 functions in alkaline tolerance mainly via alleviating oxidative damage

The common feature for plants grown in calcareous soils is chlorosis, a typical symptom of Fe deficiency. Therefore, studies on the improvement of tolerance to the stress focused mostly on the enhancement of Fe uptake in the transgenic plants [8]–[14]. Although *alt1* showed stable capacity of metal ion absorption including Fe under alkaline stress, none of the genes involved in metal ion utilization was differentially expressed between the mutant and WT (Table S2). In fact, whole genome transcriptional profiling of soybean under Fe deficiency [52] and *Puccinellia tenuiflora* under alkaline salt stress [15] revealed that the differentially expressed genes comprise various functional groups, including those related to ROS homeostasis and DNA repair. Besides, study of glutathione reductases, a large family of enzymes functioning in scavenging of ROS [50], in graminaceous plants found that expression of the related coding genes could be induced upon Fe deficiency [53], suggesting the involvement of ROS homeostasis in Fe-deficiency response. However, no direct evidence so far has been shown to connect ROS homeostasis with plant alkaline tolerance. Using microarray analysis, we found that transcription of 78 abiotic stress-related genes was significantly altered in the mutant background, with 50 out of them involving in oxidative stress response (Figure 7 and Table 1). Consistently, the mutant showed low levels of H_2_O_2_ accumulation and tolerance to oxidative stress (Figure 8). According to the report by Møller (2001) [54], strategies adopted by the plants to defend against oxidative stress include three lines of steps, i.e., avoid of ROS production, detoxification of ROS (ROS scavenging), and repair of ROS-mediated damage. The detected 50 oxidative stress-related genes in our study covered all the above three lines of defense steps (Table 1), suggesting the importance of *ALT1* in defending against the oxidative damage induced by alkaline stress.

Despite much progress, it still remains unclear how ROS affect the stress response of plants. In general, ROS is thought to affect stress responses by two different means [52], [55]. First, ROS react with cell structures, nucleic acids, lipids, and proteins [55], and may thus cause irreversible damage that can lead to tissue necrosis and ultimately plant death. Second, ROS influence the expression of a number of genes and signal transduction pathways, which activate and control various genetic stress response programs [52], [55]. Our results presented here revealed that ROS homoeostasis plays a critical regulatory role in the plant's tolerance to alkaline stress, which might involve both of the means described above. The most significant effect of the *alt1* mutation on the plant's tolerance to alkaline stress is the reduction of ROS levels, which protects the roots of the mutant from damaging oxidative stress (Figure 6). On the other hand, the differentially expressed genes detected in our microarray assay (Table S2) suggest that the induced ROS may trigger alkaline stress responses in *alt1* by modulating gene expression in an indirect manner. For example, except for the genes related to oxidative stress defense, many kinds of transcription factors involved in the abiotic stress response, such as WRKY, MYB, NAC, and bZIP, were found to be differentially regulated in the mutant (Table S2 and Figure S4). Furthermore, a series of protein kinases, including MAPK, was also identified in the microarray analysis (Table S2). Previous studies suggested that ROS generation induced by various stresses activates MAPK signaling cascades, and ROS-induced activation of MAPKs appears to be central for mediating cellular responses to multiple stresses [55]. The broadly affected genes by *ALT1* suggest that the role of ROS in regulating tolerance to alkaline stress is complicated and involves many processes.

Although the enhanced defense against oxidative damage in *alt1* gave plants tolerant to alkaline stress (Figure 1), the mutant showed similar response to drought and salt as WT (Figure S2). This observation suggests that the effects of oxidative stress might be different among abiotic stress conditions. So far, many studies have shown that ROS is involved in the response of plants to various abiotic stresses including drought and salt [56]. For example, mutation in *ITN1* increased tolerance of plants to salt stress by enhancing ROS scavenging, but the mutant displayed a WT response when under drought conditions [57]. In addition, the reduction in scavenging of ROS resulted in *dsm1* and *dsm2* hypersensitive to drought stress, but the two mutants showed similar response as WT under cold or heat stress conditions [51], [58]. Furthermore, previous studies have shown that the oxidative stress induced by Fe deficiency is fundamentally different from that induced by other factors such as salt or heavy metal stress [53], [59]. Therefore, the differential responses of *alt1* to alkaline and drought or salt observed in our study (Figure 1A and Figure S2) also support this notion that the oxidative stress induced by alkaline might be different from that induced by salt or drought. The cause for such difference, however, remained to be understood.

### ALT1 provides a novel finding on the involvement of chromatin remodeling ATPase in plant alkaline tolerance

Chromatin remodeling plays a central role in establishing specific gene expression patterns and maintaining transcriptional states in eukaryotes [24], and loss of function of the component proteins usually results in alteration in multiple plant developmental processes [25]. Furthermore, emerging evidence shows that chromatin regulation also plays important functions in plant abiotic stress responses [26]. Interestingly, most of the core subunit ATPase of chromatin remodeling complex, such as AtCHR12 [27] and AtBRM [28] in *Arabidopsis*, function as negative regulators in abiotic stress tolerance. The rice genome contains 40 Snf2 family proteins [29]. However, only one member of OsCHR4 was reported to function in early chloroplast development [30]. Transcriptional profiling of the 40 genes found that some of the members respond to abiotic stresses such as draught, salt and cold [29]. However, how about the response of these genes to alkaline stress remained to be investigated. The ALT1 protein identified here belongs to the Ris1 subgroup of Snf2 family [29]. Similar to the various functions found for the Snf2 family ATPases [25], knockout *alt1* plants displayed multiple morphological alterations, including reduced root length and plant height at early vegetative stage and low tiller number at adult stage (Figure 2C and Table S1). However, unlike the function of any other Snf2 family proteins reported so far [25], ALT1 functions negatively in stress tolerance to alkaline but not salt or drought (Figure 1 and Figure S2), consistent with the report that *ALT1* was excluded from the Snf2 family genes that show transcriptional response to the stresses of salt and drought [29]. Therefore, our results provided a novel finding on the involvement of chromatin remodeling complex in alkaline stress response. The modulation of the expression of several batches of genes (Table S2) suggests that *ALT1* might be a key regulator that occupies a critical site in the gene transcriptional regulatory network by modulating chromatin structure. However, we still have much to learn about the molecular mechanisms of the chromatin remodeling complexes and their biochemical capabilities [25], and further studies need to be performed to reveal the molecular function of ALT1 in alkaline stress tolerance.

### ALT1 provides a potential two-step strategy for improving the tolerance of rice to severe alkaline stress

The underground part of roots is the first organ to perceive most of the abiotic stresses such as drought, salt or alkaline. Although the roots could also signal the stress to the aerial parts of the plants via various pathways [60], its basic function of water and nutrient absorption is indispensable for any other parts of the plant. However, almost all these stresses would inevitably induce excess accumulation of ROS within the plants when under severe stress conditions, and the over-accumulated ROS would react with cell structures, nucleic acids, lipids and proteins, which ultimately lead to tissue necrosis and plant death [50]. Therefore, although the low Fe availability is considered to be the main reason for the reduced crop yield and quality on calcareous soils [8], [9], a survey of the related reports revealed that almost all of the researches were conducted under the mild alkaline condition of pH 8.5 [8]–[14]. This could be due to the fact that under alkaline stress higher than pH 9.0, cell death would occur rapidly in the roots of rice plants, as that observed for WT roots in our study (Figure 6). Therefore, it would be of little effect to change the insoluble Fe into an absorbable form for plants without functional roots under such alkaline conditions. On the other hand, although mutation in *ALT1* could keep the roots of the plants functionally in nutrient uptake (Figure 5), the mutant could not survive the alkaline stress of pH 9.5 until adult stage (data not shown). This could be explained by that only a very limited absorbable free form of Fe^2+^ could be utilized under such conditions [32], [61], [62], which is far from sufficient for developmental, physiological and biochemical processes of the growing *alt1* plants. From these points of view, the strategy for genetic improvement of rice plants tolerant to severe alkaline stress should combine both steps described above, i.e., the first step of utilizing mutated form of *ALT1* to ensure normal root function by alleviating oxidative damage, and the second step of exploiting genes to increase the production and secretion of mugineic acid family phytosiderophores to chelate the large amount of oxidized form of Fe^3+^ [32]. Verification of this hypothesis is now underway.

## Materials and Methods

### Plant materials and growth conditions

The rice mutant *alt1* is in the KY131 background. Rice plants were cultivated in an experimental field at the Institute of Genetics and Developmental Biology (IGDB) in Beijing. An F_2_ mapping population was constructed by crossing *alt1* (*Japonica*) with Kasalath (*Indica*). Rice plants used for the experiments were cultured in Kimura's culture solution B with the following composition: 0.18 mM (NH_4_)_2_SO_4_, 0.27 mM Mg(SO_4_)_2_, 0.091 mM KNO_3_, 0.091 mM KH_2_PO_4_, 0.046 mM K_2_SO_4_, 0.18 mM Ca(NO_3_)_2_, and 0.04 mM EDTA-Fe, and grown under natural conditions.

### Stress treatment

For alkaline stress treatment, seedlings of two-leaf stage WT, *alt1*, and RNAi transgenic plants were transferred to Kimura's culture solution B, and the solution was adjusted to pH 9.0 by adding 25 mM NaHCO_3_ and 1.765 mM NaOH, to pH 9.5 by adding 25 mM NaHCO_3_ and 5 mM NaOH, and to pH 10.0 by adding 25 mM NaHCO_3_ and 10.7 mM NaOH. For other stresses, two-leaf stage *alt1* and WT seedlings were subjected to Kimura's culture solution B with the addition of 80, 120, 150, and 180 mM NaCl for salt treatment, and 20 µM methyl viologen (MV) for oxidative stress treatment, respectively. These solutions were renewed every 3 days. For drought treatment, *alt1* and WT seedlings were grown to the two-leaf stage in pots filled with nutrient soil, and water was withheld to allow drought stress to develop for 5, 6 and 7 days before irrigation, respectively.

For investigation of transcriptional response of *ALT1* to alkaline stress, roots of two-leaf-stage WT seedlings under alkaline stress of pH 10.0 were sampled at 0, 1, 4, 12 and 24 hours after the start of treatment, respectively.

### Map-based cloning

The *alt1* locus was firstly mapped between markers RM11740 and RM3285 on chromosome 1 using 316 F_2_ mutant plants derived from cross of *alt1* with Kasalath. For fine mapping, two other molecular markers were developed and the underlying gene was further narrowed down to a 35.2-kb region between markers M1 and M6 using 538 F_2_ mutant plants. Primers used in this study are listed in Table S3. Three predicted ORFs within this region were then sequenced and compared with WT and Nipponbare (http://rapdb.dna.affrc.go.jp/).

### Plasmid construction and transgenic experiments

For complementation test, the full-length coding sequence of *Os01g0779400* driven by its native promoter (1.1-kb region upstream of the ATG start codon) was ligated into the binary vector pZH2B, and the plasmid was then transformed into *Agrobacterium tumefaciens* AGL-1. Plant transformation was conducted as described previously [51].

The *ALT1*-RNAi construct was generated by inserting a hairpin sequence with two 367-bp cDNA inverted repeats targeting the sequence from 3516–3882 bp downstream of the start codon of *ALT1*. The 367-bp fragment was ligated into pZH2Bi and driven by the *Ubiquitin* promoter. ZH11 (*Japonica*) was used as the receptor plant for transformation by *Agrobacterium*. Primers used for vector construction are listed in Table S3.

### ALT1 subcellular localization

To investigate the subcellular localization of ALT1, the *Ubi::ALT1-sGFP* plasmid and empty vector were introduced into onion epidermal cells using particle bombardment with a PDS-1000/He (BIO-RAD). After overnight incubation at 25°C in darkness, bombarded tissues were examined by confocal laser-scanning microscopy (Leica TCS SP5) using 488-nm excitation and 500- to 530-nm emissions pass filters.

### 3,3′-diaminobenzidine (DAB) and propidium iodide (PI) staining

DAB staining was carried out by the method of Ning et al. (2010) [51], and PI staining was performed as described previously [35] with slight modifications. Briefly, roots of seedlings were immersed in the solution containing 1 µg mL^−1^ PI in distilled water, incubated for 1 min at room temperature, and then washed with 1× phosphate buffered saline (PBS) several times. Staining was observed using fluorescence microscopy (OLYMPUS DP72).

### Metal ion concentration measurement

Shoots of two-leaf stage *alt1* and WT seedlings were collected at day 0, 3, 6, and 10 after alkaline stress treatment (pH 9.5), and dried in a 65°C incubator for 1 week. The samples (100–150 mg) were then wet-ashed with 13 mL of 11 M HNO_3_ and 2 mL of 30% H_2_O_2_ according to the following procedure: 110°C for 10 min, 130°C for 10 min, 150°C for 15 min, and 150°C for 60 min. The metal ion concentration was measured using an Inductively Coupled Plasma-Optical Emission Spectrometer (ICP-OES, Perkin Elmer, USA) at wavelengths of 238.204 nm (Fe), 279.077 nm (Mg), 327.393 nm (Cu), 206.200 nm (Zn), and 257.610 nm (Mn), respectively, with three independent biological replicates.

### Microarray analysis

Roots of two-leaf stage *alt1* and WT seedlings grown under normal conditions were sampled for microarray analysis. The transcriptomic profiles were investigated using an Agilent-015241 Rice Gene Expression 4×44 K Microarray (Agilent Technology) containing 32,325 probes corresponding to cDNA, 6,934 probes corresponding to expressed sequence tags (ESTs), and 2,612 probes corresponding to gene predicted loci, respectively, with three independent biological replicates. All microarray procedures and data analyses were performed according to the manufacturer's instructions. The microarray data were deposited in Gene Expression Omnibus of NCBI under accession number: GSE61788 (http://www.ncbi.nlm.nih.gov/geo/info/linking.html). Analysis was performed using an ANOVA-false discovery rate (ANOVA-FDR) p-value of <0.01. Spots with changes in expression were extracted based on at least a threefold increase or decrease in expression. Functional classification of the differentially expressed genes was carried out using the tools for GO categories and revised manually.

### Quantitative real-time RT-PCR (qRT-PCR) analysis

Total RNA was extracted using a TaKaRa RNAiso Plus Kit. For qRT-PCR, the RNA was pre-treated with DNase I (Fermentas), and first-strand cDNA was synthesized from 1 µg total RNA according to manufacturer's protocol (Promega). qRT-PCR was performed with the Lightcycler 480 SYBR Green I Master according to the manufacturer's protocol. Three repeats were carried out for each gene. For normalization, the *Actin* gene was used as the endogenous control. Primers used for qRT-PCR are listed in Table S3.

### H_2_O_2_ measurement

The level of H_2_O_2_ was assessed as described [63]. Briefly, 200 mg of fresh leaf tissue from *alt1* and WT seedlings subjected to alkaline stress treatment was extracted in 2.0 ml of TCA (0.1% w/v) on ice, and the homogenate was then centrifuged at 13,000 g for 15 min. After the addition of 0.3 ml of 10 mM sodium phosphate buffer (pH 7.5) and 0.6 ml of 1 M potassium iodide to 0.3 ml of the above supernatant, the absorbance of the samples was measured at 390 nm. H_2_O_2_ content, expressed as nmol g^−1^ fresh weight, was determined based on the standard curve generated from known concentrations of H_2_O_2_.

## Supporting Information

Figure S1 **Phenotypic analysis of the** ***alt1*** **mutant.** Two-leaf stage WT (left) and *alt1* (right) seedlings were subjected to alkaline treatment with pH 9.0, and photographed at 15 days after treatment.
(TIF)

Figure S2 **The** ***alt1*** **mutant showed a normal response to salt and drought stresses.** Left part: WT; Right part: *alt1*. Two-leaf stage *alt1* and WT seedlings were subjected to NaCl and drought treatments, respectively.
(TIF)

Figure S3 **Comparison of the predicted amino acid sequence of ALT1 and the truncated alt1 protein in the mutated region.** The amino acids of the mutated region from 720 aa to 1228 aa of ALT1 are shown. The truncated alt1 stopped at 1085 aa.
(TIF)

Figure S4 **Expression analysis of selected TF genes.** qRT-PCR was conducted on the roots of hydroponically grown two-leaf stage *alt1* and WT seedlings. Actin was used as an internal control. Data shown are mean values of three biological repeats with SD.
(TIF)

Table S1 **Comparison of agronomic traits between** ***alt1*** **and WT.**
(DOCX)

Table S2 **List of genes up/down-regulated 3 fold more in** ***alt1*** **as revealed by microarray analysis.**
(DOCX)

Table S3 **Primers used in this study.**
(DOCX)
