# Supplementary figures and images for: ALT1, a Snf2 Family Chromatin Remodeling ATPase, Negatively Regulates Alkaline Tolerance through Enhanced Defense against Oxidative Stress in Rice
Source: PLoS One. 2014 Dec 4;9(12):e112515. doi: 10.1371/journal.pone.0112515 (PMC4256374; doi:10.1371/journal.pone.0112515)

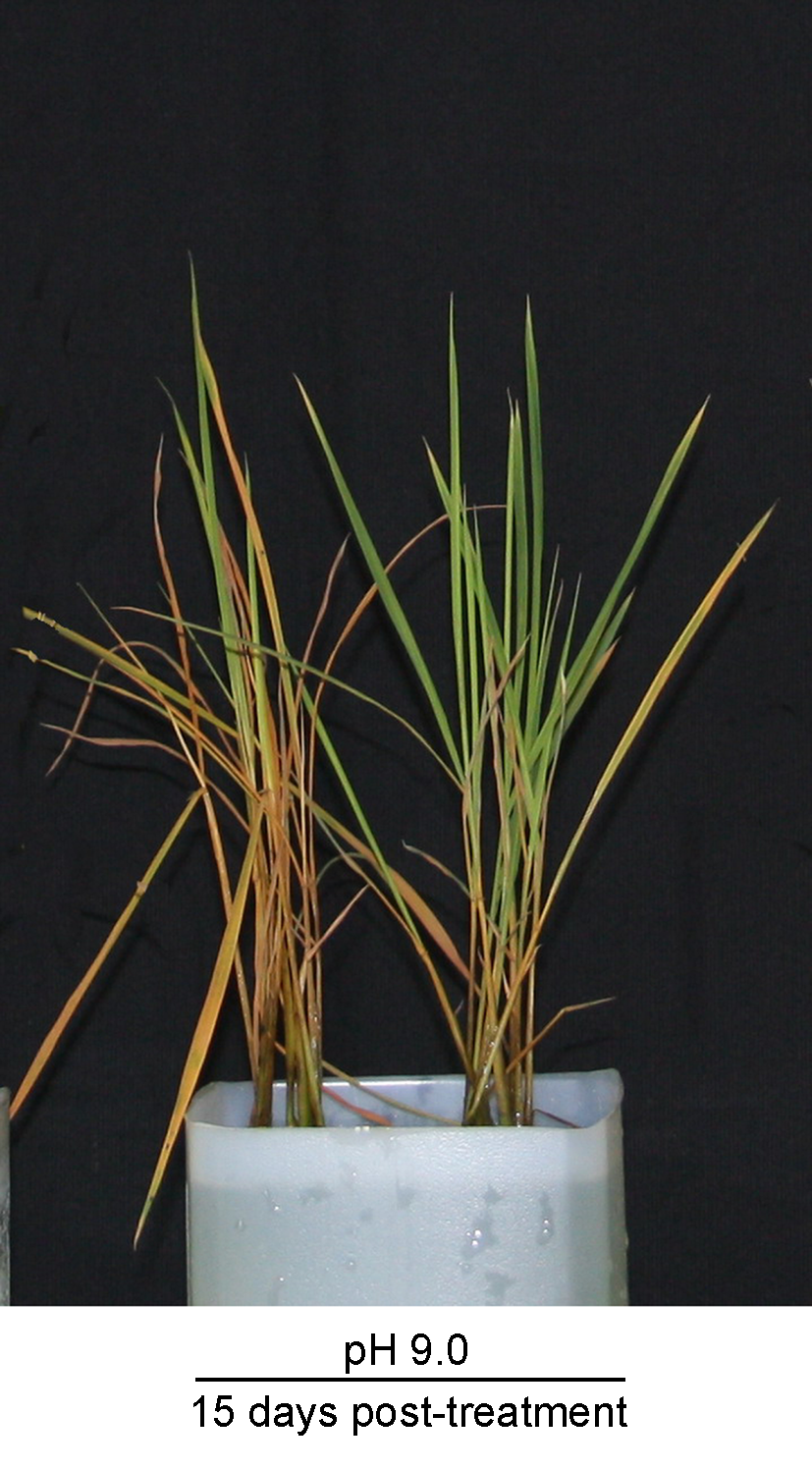

Supplement: Figure S1 — Phenotypic analysis of the alt1 mutant. Two-leaf stage WT (left) and alt1 (right) seedlings were subjected to alkaline treatment with pH 9.0, and photographed at 15 days after treatment. (TIF) [file pone.0112515.s001.tif]

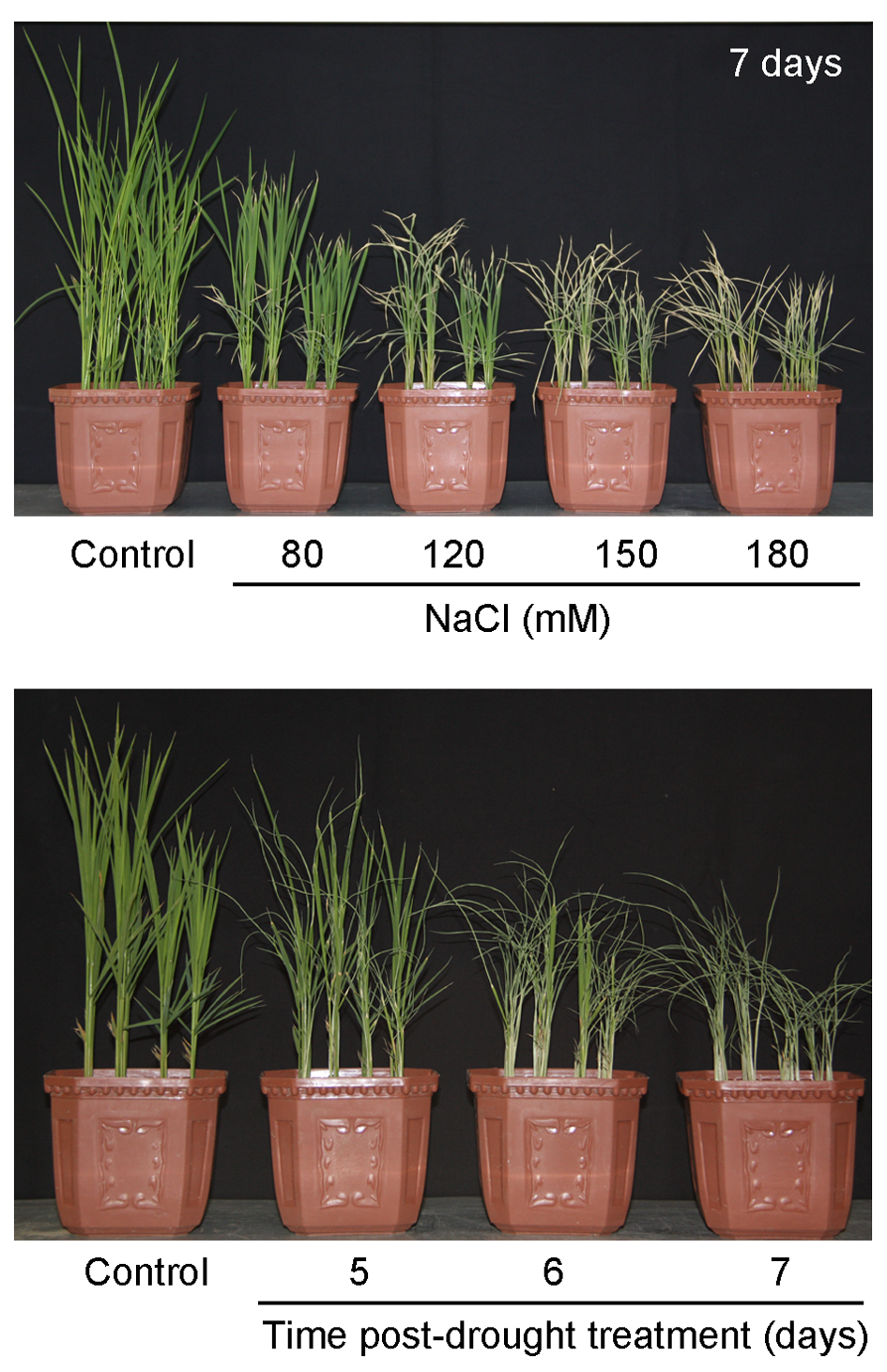

Supplement: Figure S2 — The alt1 mutant showed a normal response to salt and drought stresses. Left part: WT; Right part: alt1. Two-leaf stage alt1 and WT seedlings were subjected to NaCl and drought treatments, respectively. (TIF) [file pone.0112515.s002.tif]

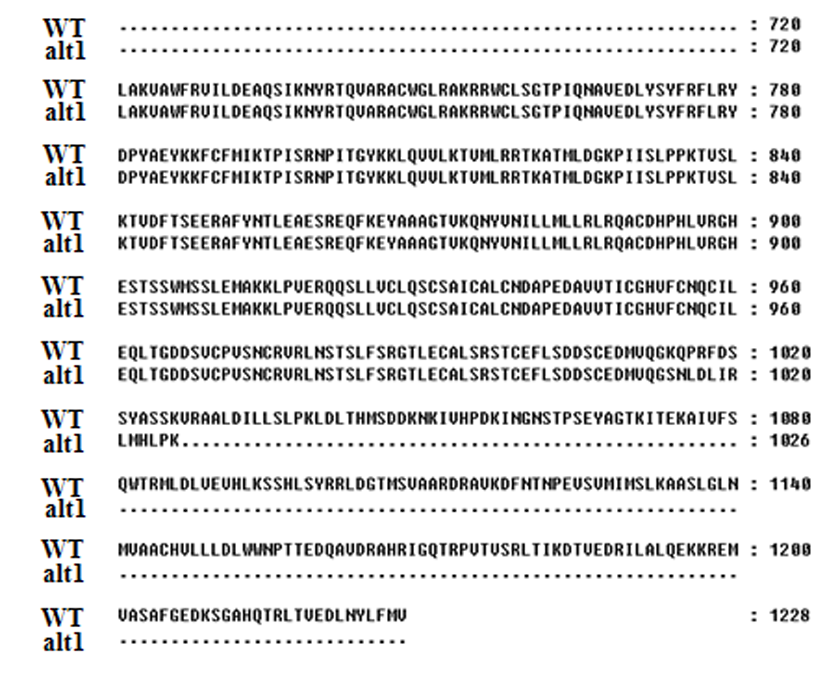

Supplement: Figure S3 — Comparison of the predicted amino acid sequence of ALT1 and the truncated alt1 protein in the mutated region. The amino acids of the mutated region from 720 aa to 1228 aa of ALT1 are shown. The truncated alt1 stopped at 1085 aa. (TIF) [file pone.0112515.s003.tif]

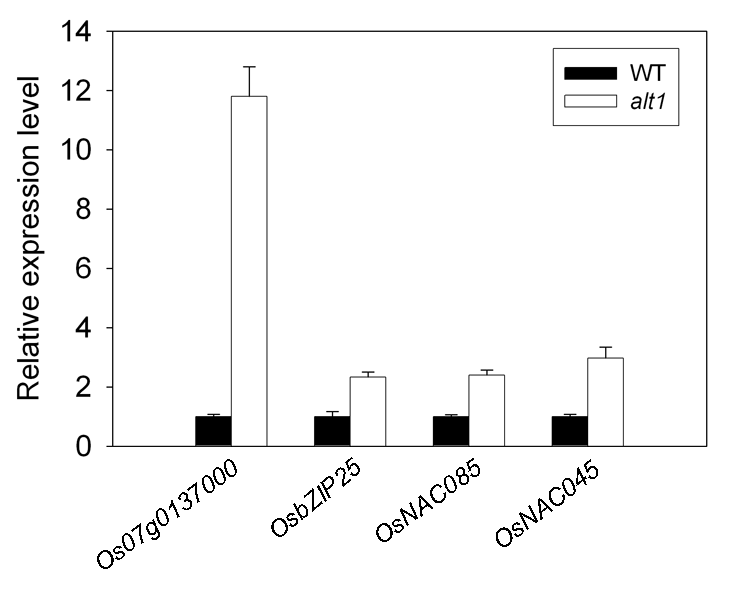

Supplement: Figure S4 — Expression analysis of selected TF genes. qRT-PCR was conducted on the roots of hydroponically grown two-leaf stage alt1 and WT seedlings. Actin was used as an internal control. Data shown are mean values of three biological repeats with SD. (TIF) [file pone.0112515.s004.tif]
