# Supplementary material for: ALT1, a Snf2 Family Chromatin Remodeling ATPase, Negatively Regulates Alkaline Tolerance through Enhanced Defense against Oxidative Stress in Rice
Source: PLoS One. 2014 Dec 4;9(12):e112515. doi: 10.1371/journal.pone.0112515 (PMC4256374; doi:10.1371/journal.pone.0112515)
Supplement: Table S1 — Comparison of agronomic traits between alt1 and WT. (DOCX) [file pone.0112515.s005.docx]

**Table S1. Comparison of agronomic traits between *alt1* and WT.**

| **Character** | **WT** | ***alt1*** |
| --- | --- | --- |
| **Heading date** | 12-Aug | 12-Aug |
| **Tiller number** | 9.7 ± 1.8 (20) | 6.3 ± 1.1 |
| **Plant height (cm)** | 97.3 ± 2.8 (30) | 95.9 ± 2.6 |
| **Seed setting (%)** | 95.7 ± 3.4 (8) | 93.8 ± 4.3 |
| **Thousand grain weight (g)** | 26.3 ± 1.2 (3) | 25.8 ± 1.4 |

Numbers in parenthesis indicate sample number. Values represent means ± SD.
