# Supplementary material for: ALT1, a Snf2 Family Chromatin Remodeling ATPase, Negatively Regulates Alkaline Tolerance through Enhanced Defense against Oxidative Stress in Rice
Source: PLoS One. 2014 Dec 4;9(12):e112515. doi: 10.1371/journal.pone.0112515 (PMC4256374; doi:10.1371/journal.pone.0112515)
Supplement: Table S2 — List of genes up/down-regulated 3 fold more in alt1 as revealed by microarray analysis. (DOCX) [file pone.0112515.s006.docx]

**Table S2. List of genes up/down-regulated 3 fold more in *alt1* as revealed by microarray analysis.**

| **Probe name^a^** | **Description^b^** | **P-values^c^** | **Fold change^d^** |
| --- | --- | --- | --- |
| **Transcription factors** | |  |  |
| ***Os03g0127500*^e^** | bZIP transcription factor, bZIP-1 domain containing protein | 0.000831 | 3.79 |
| ***Os05g0129800*** | DNA-binding WRKY domain containing protein | 0.001668 | 3.70 |
| ***Os01g0289600*** | Similar to WRKY transcription factor 5 | 0.002209 | -3.12 |
| ***Os09g0417800*** | WRKY transcription factor 62 | 0.008293 | -3.73 |
| ***Os09g0417600*** | WRKY transcription factor 76 | 0.000554 | -6.43 |
| *Os08g0230600* | LIM, zinc-binding domain containing protein | 0.002558 | -4.21 |
| ***Os07g0137000*** | Myb transcription factor domain containing protein | 0.001286 | 16.41 |
| ***Os01g0524500*** | Transcription factor MYBS3 | 0.000750 | -4.00 |
| ***Os04g0477300*** | No apical meristem (NAM) protein domain containing protein | 0.000294 | 3.88 |
| ***Os11g0127600*** | No apical meristem (NAM) protein domain containing protein | 0.000621 | 7.19 |
| ***Os12g0123800*** | No apical meristem (NAM) protein domain containing protein | 0.000048 | 5.95 |
| ***Os02g0594800*** | No apical meristem (NAM) protein domain containing protein | 0.000013 | 6.83 |
| ***Os05g0194500*** | Similar to ANAC075 | 0.000063 | 11.65 |
| *Os08g0438400* | Similar to ZF-HD homeobox protein | 0.002228 | -3.24 |
| *Os08g0324300* | Transcriptional factor B3 family protein | 0.000689 | 3.08 |
| *Os08g0249000* | Zinc finger, B-box domain containing protein | 0.003633 | -3.24 |
| ***Os11g0702400*** | Zinc finger, C2H2-type domain containing protein | 0.000504 | -4.08 |
| *Os01g0276600* | Zinc finger, RING/FYVE/PHD-type domain containing protein | 0.002207 | -4.56 |
| *Os01g0311400* | Zinc finger, RING/FYVE/PHD-type domain containing protein | 0.004924 | -3.75 |
| *Os06g0535200* | Zinc finger, RING/FYVE/PHD-type domain containing protein | 0.002048 | -5.70 |
| *Os05g0468900* | Zinc finger, RING/FYVE/PHD-type domain containing protein | 0.001759 | -3.10 |
| *Os09g0505000* | Zinc finger, RING/FYVE/PHD-type domain containing protein | 0.000621 | -3.73 |
| *Os01g0865700* | Zinc finger, RING/FYVE/PHD-type domain containing protein | 0.000748 | -4.22 |
| *Os04g0417400* | Zinc finger, RING/FYVE/PHD-type domain containing protein | 0.000964 | -4.76 |
| *Os04g0418500* | Zinc finger, RING/FYVE/PHD-type domain containing protein | 0.003205 | -3.44 |
| *Os04g0589700* | Zinc finger, RING/FYVE/PHD-type domain containing protein | 0.000639 | -3.36 |
| **Defence and virulence** | |  |  |
| *Os08g0480200* | 2OG-Fe(II) oxygenase domain containing protein | 0.000112 | 3.72 |
| *Os11g0657200* | 2OG-Fe(II) oxygenase domain containing protein | 0.006750 | -8.57 |
| ***Os01g0959200*** | Abscisic stress ripening protein 1 | 0.003582 | -12.29 |
| ***Os11g0210300*** | Alcohol dehydrogenase 1 | 0.000040 | 3.22 |
| ***Os04g0339400*** | Aldo/keto reductase family protein | 0.000002 | 236.55 |
| ***Os12g0226900*** | Similar to Allyl alcohol dehydrogenase | 0.000191 | 3.49 |
| ***Os02g0585700*** | Quinonprotein alcohol dehydrogenase-like domain containing protein | 0.001116 | -5.40 |
| ***Os02g0586000*** | Quinonprotein alcohol dehydrogenase-like domain containing protein | 0.000832 | -21.53 |
| ***Os05g0573200*** | NADP-isocitrate dehydrogenase | 0.001074 | 5.54 |
| ***Os05g0331200*** | External rotenone-insensitive NADPH dehydrogenase | 0.000550 | 17.33 |
| ***Os08g0141400*** | Similar to External rotenone-insensitive NADPH dehydrogenase | 0.000892 | 5.39 |
| ***Os01g0633500*** | Similar to NADPH-dependent reductase A1 | 0.002659 | -4.34 |
| *Os03g0766900* | Allene oxide synthase | 0.000216 | 3.35 |
| ***Os04g0600200*** | Alternative oxidase 1a | 0.000511 | 7.53 |
| ***Os02g0318100*** | Alternative oxidase 1a | 0.000669 | 23.84 |
| ***Os01g0801100*** | Apurinic endonuclease-redox protein (DNA-(apurinic or apyrimidinic site) lyase) | 0.000007 | 4.50 |
| *Os08g0374000* | Bet v I allergen family protein | 0.001115 | -3.28 |
| *Os01g0947000* | Beta-1,3-glucanase precursor | 0.002965 | 6.68 |
| *Os05g0535100* | Beta-1,3-glucanase-like protein | 0.000214 | 6.19 |
| ***Os01g0939300*** | BRCT domain containing protein | 0.000063 | 5.44 |
| *Os02g0148000* | CCT domain containing protein | 0.002749 | -3.13 |
| *Os01g0860500* | Chitinase (EC 3.2.1.14) | 0.001592 | -3.88 |
| ***Os09g0370200*** | Copper chaperone SCO1/SenC domain containing protein | 0.000104 | 4.78 |
| ***Os02g0791400*** | Cytochrome c oxidase, subunit VIb domain containing protein | 0.008472 | -3.26 |
| ***Os08g0496000*** | Cytochrome oxidase assembly family protein | 0.002097 | 3.12 |
| ***Os05g0498300*** | DNA mismatch repair protein MutS, core domain containing protein | 0.000005 | 3.71 |
| ***Os07g0209500*** | DNA-directed DNA polymerase, family B domain containing protein | 0.001388 | 5.03 |
| ***Os03g0776900*** | DNAJ protein-like | 0.000593 | 10.31 |
| ***Os09g0284400*** | GrpE nucleotide exchange factor domain containing protein | 0.000002 | 5.13 |
| ***Os02g0758000*** | Low molecular weight heat shock protein precursor | 0.000003 | 10.19 |
| ***Os05g0364500*** | Similar to Chaperone protein DNAJ | 0.004105 | -3.20 |
| ***Os02g0181900*** | Similar to ClpB | 0.000054 | 5.77 |
| ***Os02g0794600*** | Similar to Copper chaperone COX17-1 | 0.000085 | 7.34 |
| ***Os06g0219500*** | Similar to Heat shock 22 kDa protein, mitochondrial precursor | 0.000235 | 13.00 |
| *Os07g0625600* | Fimbriata-associated protein | 0.000389 | 10.78 |
| *Os05g0135000* | Haem peroxidase family protein | 0.006922 | -3.99 |
| *Os03g0339400* | Haem peroxidase, plant/fungal/bacterial family protein | 0.003005 | 3.20 |
| *Os07g0104600* | Haem peroxidase, plant/fungal/bacterial family protein | 0.000809 | -5.50 |
| *Os04g0688300* | Haem peroxidase, plant/fungal/bacterial family protein | 0.000448 | -4.21 |
| *Os07g0638800* | Similar to Peroxidase | 0.000125 | -10.82 |
| *Os07g0638900* | Similar to Peroxidase | 0.001662 | -10.29 |
| *Os07g0626700* | Similar to Peroxidase | 0.004351 | -3.40 |
| *Os07g0638600* | Peroxidase 1 | 0.001571 | -5.68 |
| *Os01g0962700* | Peroxidase 12 precursor | 0.000094 | -4.92 |
| *Os11g0307300* | Herbicide safener binding protein | 0.002014 | 6.77 |
| *Os10g0161400* | NB-ARC domain containing protein | 0.000931 | 3.13 |
| *Os11g0605100* | NB-ARC domain containing protein | 0.001893 | -3.44 |
| *Os11g0161000* | NB-ARC domain containing protein | 0.003505 | -3.41 |
| ***Os06g0618000*** | Nse4 domain containing protein | 0.000007 | 7.02 |
| *Os08g0189500* | Oryza sativa germin-like protein 8-6 | 0.008654 | -3.89 |
| *Os12g0611300* | Patatin family protein | 0.000472 | -3.54 |
| *Os08g0450100* | Pectinesterase (EC 3.1.1.11) | 0.009986 | 3.55 |
| ***Os07g0138100*** | Pleckstrin homology-type domain containing protein | 0.000420 | -5.29 |
| *Os10g0546100* | Pollen Ole e 1 allergen and extensin domain containing protein | 0.000198 | -14.63 |
| ***Os10g0550900*** | Proline oxidase domain containing protein | 0.000020 | -6.01 |
| ***Os05g0580500*** | Rad21/Rec8 like protein, N-terminal domain containing protein | 0.000034 | 7.21 |
| *Os01g0894500* | Sep15/SelM redox domain containing protein | 0.000033 | -3.87 |
| ***Os06g0216300*** | Similar to 12-oxophytodienoic acid reductase | 0.005153 | 4.12 |
| *Os02g0609200* | Similar to ATOZI1 protein | 0.000008 | 3.55 |
| *Os03g0114400* | Similar to Avr9/Cf-9 rapidly elicited protein 102 | 0.001641 | -5.08 |
| *Os02g0818000* | Similar to Brown planthopper-induced resistance protein 1 | 0.004698 | -3.01 |
| *Os05g0399300* | Similar to Chitinase | 0.008603 | -4.58 |
| *Os10g0416500* | Similar to Chitinase 1 precursor | 0.005500 | -4.21 |
| *Os02g0629800* | Similar to Defensin precursor | 0.003705 | -4.10 |
| ***Os11g0146800*** | Similar to Disrupted meiotic cDNA 1 protein | 0.001043 | 4.79 |
| ***Os12g0143800*** | Similar to Disrupted meiotic cDNA 1 protein | 0.000016 | 8.18 |
| ***Os12g0497300*** | Similar to DNA repair protein RAD51 homolog | 0.000015 | 8.34 |
| ***Os09g0381400*** | Similar to Ervatamin C | 0.000013 | 4.33 |
| *Os07g0271000* | Similar to GDP dissociation inhibitor protein OsGDI1 | 0.002006 | 3.07 |
| ***Os03g0595600*** | Similar to Glutathione S-transferase | 0.000001 | 9.45 |
| ***Os01g0692100*** | Glutathione S-transferase, C-terminal-like domain containing protein | 0.000003 | 46.22 |
| ***Os10g0528400*** | Glutathione S-transferase, C-terminal-like domain containing protein | 0.000071 | 11.62 |
| ***Os01g0949900*** | Glutathione S-transferase, C-terminal-like domain containing protein | 0.000007 | 35.86 |
| ***Os01g0692000*** | Similar to Glutathione S-transferase GST 26 | 0.000001 | 22.47 |
| ***Os01g0949800*** | Similar to Glutathione S-transferase GST 28 | 0.000001 | 6.75 |
| ***Os01g0950000*** | Similar to Glutathione S-transferase GST 28 | 0.000352 | 11.56 |
| ***Os10g0530900*** | Similar to Glutathione S-transferase GST 30 | 0.004421 | 6.11 |
| ***Os05g0412800*** | Similar to Glutathione S-transferase GST 41 | 0.000014 | 20.62 |
| ***Os01g0369700*** | Similar to Glutathione S-transferase GST 8 | 0.001745 | 3.00 |
| ***Os10g0525800*** | Similar to Glutathione S-transferase GSTU31 | 0.008134 | 3.25 |
| ***Os10g0368100*** | Similar to Glutathione S-transferase GSTU35 | 0.000444 | 23.42 |
| ***Os10g0528200*** | Similar to Glutathione S-transferase TSI-1 | 0.000126 | 4.63 |
| ***Os01g0371200*** | Similar to Glutathione-S-transferase 19E50 | 0.001183 | 3.87 |
| ***Os05g0148900*** | Similar to Glutathione-S-transferase 19E50 | 0.001472 | -3.29 |
| ***Os10g0529500*** | Similar to Glutathione-S-transferase 2 | 0.000013 | 3.23 |
| ***Os03g0643700*** | Similar to GST6 protein | 0.002487 | -8.45 |
| ***Os09g0367700*** | Similar to GST6 protein | 0.000059 | 25.58 |
| ***Os10g0527400*** | Similar to Tau class GST protein 3 | 0.000101 | 58.60 |
| ***Os10g0525600*** | Similar to Tau class GST protein 3 | 0.000019 | 42.78 |
| ***Os10g0528300*** | Tau class GST protein 4 | 0.000161 | 36.84 |
| ***Os10g0529400*** | Tau class GST protein 4 | 0.002258 | 3.23 |
| ***Os01g0106400*** | Similar to Isoflavone reductase homolog IRL | 0.000257 | 14.03 |
| *Os12g0596000* | Similar to Lipoyltransferase | 0.000235 | -3.26 |
| ***Os10g0462900*** | Similar to mitochondrial chaperonin-60 | 0.004277 | 4.25 |
| *Os08g0170700* | Similar to NB-ARC domain containing protein, expressed | 0.004095 | -20.61 |
| *Os12g0281300* | Similar to NB-ARC domain containing protein, expressed | 0.003106 | -10.16 |
| *Os02g0281200* | Similar to NBS-LRR protein | 0.001468 | 3.44 |
| *Os01g0781100* | Similar to NBS-LRR protein | 0.001437 | -3.03 |
| *Os01g0194300* | Similar to NPR1 | 0.005308 | -3.47 |
| *Os10g0490900* | Similar to NtPRp27 | 0.004910 | -3.74 |
| *Os01g0369900* | Similar to Oxo-phytodienoic acid reductase | 0.001883 | -3.41 |
| *Os06g0216200* | Similar to Oxo-phytodienoic acid reductase | 0.002715 | 3.97 |
| *Os12g0555300* | Similar to Pathogenesis-related protein PR-10a | 0.007124 | -3.06 |
| *Os12g0448900* | Similar to Pathogen-inducible alpha-dioxygenase | 0.001629 | 3.99 |
| *Os12g0555100* | Similar to Probenazole-inducible protein PBZ1 | 0.000582 | -4.46 |
| ***Os10g0419400*** | Similar to SIPL | 0.000010 | 3.99 |
| *Os05g0370300* | Similar to Src2-like protein | 0.000031 | -4.19 |
| *Os03g0663400* | Thaumatin-like protein | 0.000003 | -5.83 |
| ***Os01g0710700*** | Lipase, class 3 family protein | 0.007764 | 0.33 |
| ***Os05g0153300*** | Lipase, class 3 family protein | 0.000036 | -4.54 |
| ***Os01g0214800*** | Lipase, GDSL domain containing protein | 0.005987 | -3.01 |
| ***Os01g0216400*** | Lipase, GDSL domain containing protein | 0.000090 | -3.40 |
| ***Os04g0507700*** | Lipase, GDSL domain containing protein | 0.000168 | 3.68 |
| **Transporters** |  |  |  |
| *Os07g0558000* | ABC-1 domain containing protein | 0.000756 | 4.40 |
| *Os12g0485600* | Amino acid transporter, transmembrane domain containing protein | 0.000501 | 3.29 |
| *Os06g0560000* | Ferroportin1 family protein | 0.000004 | -9.69 |
| *Os11g0135900* | Major facilitator superfamily protein | 0.002429 | 3.02 |
| *Os04g0453300* | Major facilitator superfamily protein | 0.000168 | -5.02 |
| *Os10g0397800* | Mitochondrial carrier protein family protein | 0.000023 | -7.59 |
| *Os04g0524600* | Oligopeptide transporter OPT superfamily protein | 0.002390 | -3.24 |
| *Os02g0112600* | Similar to BCH2 | 0.000305 | -5.56 |
| *Os02g0306100* | Similar to Cation cation antiporter | 0.000324 | -10.22 |
| *Os08g0535000* | Similar to Cation cation antiporter | 0.000024 | -42.76 |
| *Os01g0209800* | Similar to Cationic amino acid transporter | 0.000165 | -3.86 |
| *Os10g0573700* | Similar to Mitochondrial carnitine/acylcarnitine carrier-like protein | 0.000731 | -3.39 |
| *Os02g0573500* | Similar to Monosaccharide transporter 1 | 0.000017 | -4.20 |
| *Os04g0452600* | Similar to Monosaccharide transporter 1 | 0.000445 | -4.23 |
| *Os04g0452700* | Similar to Monosaccharide transporter 1 | 0.000090 | -4.27 |
| *Os04g0453200* | Similar to Monosaccharide transporter 1 | 0.000444 | -4.89 |
| *Os02g0574000* | Similar to Monosaccharide transporter 1 | 0.002392 | -5.62 |
| *Os01g0695800* | Similar to Multidrug resistance protein 1 homolog | 0.000007 | 60.00 |
| *Os01g0533900* | Similar to Multidrug resistance protein 1 homolog | 0.001545 | 8.32 |
| *Os06g0581000* | Similar to Nitrate transporter NTL1 | 0.001984 | -3.03 |
| *Os02g0528900* | Similar to PDR9 ABC transporter | 0.001725 | 3.10 |
| *Os01g0142800* | Similar to Peptide transporter | 0.000502 | -3.03 |
| *Os02g0580900* | Similar to Peptide transporter PTR2 | 0.003575 | -3.00 |
| *Os05g0431700* | Similar to Peptide transporter PTR2-B | 0.000208 | -3.33 |
| *Os10g0360100* | Similar to Sugar transporter protein | 0.000387 | -6.28 |
| *Os10g0580400* | Similar to Urea active transporter-like protein | 0.000063 | -4.59 |
| *Os09g0484900* | Sodium/sulphate symporter family protein | 0.001657 | -3.81 |
| *Os07g0151200* | Sugar/inositol transporter domain containing protein | 0.000184 | -4.58 |
| **Protein synthesis and translation** | |  |  |
| *Os03g0694800* | Ribosomal protein L18/L5 domain containing protein | 0.000015 | -4.66 |
| *Os05g0490800* | Similar to Proteasome subunit alpha type | 0.000004 | 3.68 |
| *Os08g0529100* | Similar to Proteasome subunit beta type 1 | 0.000000 | 12.75 |
| *Os04g0627900* | Translation initiation factor SUI1 domain containing protein | 0.000144 | -3.49 |
| **Protein fate** |  |  |  |
| *Os01g0823900* | Arm repeat protein | 0.000710 | -3.98 |
| *Os07g0625400* | BTB/POZ fold domain containing protein | 0.000155 | 16.46 |
| *Os12g0594600* | BTB/POZ fold domain containing protein | 0.000502 | 13.58 |
| *Os01g0339600* | Proteasome assembly chaperone 3 domain containing protein | 0.003763 | -3.61 |
| *Os10g0395500* | Proteasome assembly chaperone 3 domain containing protein | 0.000685 | -4.51 |
| *Os04g0450300* | RING finger protein 13 (C-RZF) | 0.001280 | -3.28 |
| *Os05g0439400* | Similar to Arm repeat containing protein | 0.001267 | -3.59 |
| *Os06g0650100* | Similar to Polyubiquitin gene | 0.000003 | -4.51 |
| *Os04g0580400* | Similar to Ubiquitin-conjugating enzyme E2 I | 0.000055 | 3.13 |
| **Cellular communication or signal transduction mechanism** | |  |  |
| *Os12g0261300* | Armadillo-like helical domain containing protein | 0.000490 | -3.23 |
| *Os03g0362200* | Armadillo-like helical domain containing protein | 0.000162 | -5.09 |
| *Os02g0816300* | Armadillo-type fold domain containing protein | 0.000022 | 3.09 |
| *Os09g0309200* | Calmodulin binding protein-like family protein | 0.000353 | -3.07 |
| *Os02g0743800* | CS domain containing protein | 0.000353 | 7.39 |
| *Os09g0324400* | Cyclin-like F-box domain containing protein | 0.000181 | 4.10 |
| *Os11g0208400* | Cyclin-like F-box domain containing protein | 0.000316 | 3.14 |
| *Os12g0592500* | Cyclin-like F-box domain containing protein | 0.000645 | -3.12 |
| *Os02g0671100* | Cyclin-like F-box domain containing protein | 0.001488 | -3.68 |
| *Os11g0633800* | Cyclin-like F-box domain containing protein | 0.000011 | -4.06 |
| *Os11g0155100* | Cyclin-like F-box domain containing protein | 0.002092 | -5.04 |
| *Os02g0630000* | Cyclin-like F-box domain containing protein | 0.000437 | -5.08 |
| *Os08g0460600* | Cytokinin dehydrogenase 1, FAD and cytokinin binding domain containing protein | 0.002057 | -3.71 |
| *Os09g0458800* | EF hand domain containing protein | 0.004964 | -4.39 |
| *Os01g0765600* | EF-Hand type domain containing protein | 0.000430 | -3.46 |
| *Os03g0812400* | EF-Hand type domain containing protein | 0.001858 | -5.12 |
| *Os02g0465600* | Kelch-type beta propeller domain containing protein | 0.000645 | -3.90 |
| *Os11g0246200* | Kelch-type beta propeller domain containing protein | 0.004444 | -4.22 |
| *Os07g0546400* | NPH3 domain containing protein | 0.002638 | -3.46 |
| *Os11g0118300* | NPH3 domain containing protein | 0.000009 | -5.16 |
| *Os11g0444900* | Octicosapeptide/Phox/Bem1p domain containing protein | 0.002259 | -4.43 |
| *Os11g0444700* | Octicosapeptide/Phox/Bem1p domain containing protein | 0.001420 | -4.99 |
| *Os07g0141100* | Protein kinase, core domain containing protein | 0.000116 | 4.31 |
| *Os02g0106900* | Protein kinase, core domain containing protein | 0.001722 | -3.11 |
| *Os11g0294800* | Protein kinase, core domain containing protein | 0.000019 | -4.07 |
| *Os02g0821400* | Protein kinase, core domain containing protein | 0.000218 | -4.54 |
| *Os06g0692100* | Protein kinase, core domain containing protein | 0.001303 | -5.02 |
| *Os06g0692600* | Protein kinase, core domain containing protein | 0.000156 | -6.08 |
| *Os11g0216000* | Pyruvate kinase family protein | 0.004451 | 3.96 |
| *Os03g0113000* | Serine/threonine protein kinase domain containing protein | 0.001036 | -3.13 |
| *Os08g0376600* | Serine/threonine protein kinase domain containing protein | 0.000586 | -5.22 |
| *Os09g0454900* | Serine/threonine protein kinase-related domain containing protein | 0.001452 | -3.15 |
| *Os04g0391500* | Similar to ACR4 | 0.000055 | -5.76 |
| *Os08g0534300* | Similar to Calmodulin binding protein | 0.000001 | -13.05 |
| *Os12g0603700* | Similar to CBL-interacting protein kinase 4 | 0.004273 | -3.53 |
| *Os01g0153800* | Similar to Gibberellin receptor GID1L2 | 0.000290 | -5.83 |
| *Os11g0448300* | Similar to MAP kinase | 0.000009 | -5.33 |
| *Os09g0486700* | Similar to P90 ribosomal S6 kinase | 0.001111 | -3.41 |
| *Os03g0255500* | Similar to Phosphoenolpyruvate carboxykinase 4 | 0.000801 | -3.57 |
| *Os03g0364400* | Similar to Phytosulfokine receptor-like protein | 0.000466 | -3.29 |
| *Os01g0646300* | Similar to RGA2 protein | 0.001917 | -3.12 |
| *Os01g0206700* | Similar to Serine/threonine protein kinase | 0.001651 | 3.03 |
| *Os03g0725400* | Similar to WD-repeat protein 5 | 0.000293 | 3.98 |
| *Os04g0385600* | Tetratricopeptide-like helical domain containing protein | 0.009603 | -3.83 |
| *Os02g0635600* | Tyrosine protein kinase domain containing protein | 0.000007 | 3.56 |
| *Os04g0481600* | WD40-like domain containing protein | 0.004316 | -4.52 |
| **Development** |  |  |  |
| *Os01g0523200* | CALS5 (CALLOSE SYNTHASE 5) | 0.000018 | 4.14 |
| *Os02g0178100* | CCT domain containing protein | 0.003157 | -4.41 |
| *Os04g0497700* | CONSTANS-like protein | 0.000023 | -3.34 |
| *Os03g0132200* | Expansin-like protein A | 0.003944 | -3.66 |
| *Os04g0472200* | FAS1 domain containing protein | 0.001033 | -4.03 |
| *Os04g0415800* | Functional inhibitor/plant lipid transfer protein/seed storage domain containing protein | 0.001843 | 4.15 |
| *Os03g0233900* | Non-symbiotic hemoglobin 1 (rHb1) (ORYsa GLB1a) | 0.000142 | 11.57 |
| *Os03g0226200* | Non-symbiotic hemoglobin 2 (rHb2) (ORYsa GLB1b) | 0.005309 | 16.72 |
| *Os03g0234000* | Non-symbiotic hemoglobin 3 (rHb3) (ORYsa GLB1c) | 0.000096 | 14.35 |
| *Os02g0610500* | Oryza sativa CONSTANS-like gene 4 | 0.000388 | -3.21 |
| *Os06g0172800* | Raffinose synthase family protein | 0.000794 | -3.03 |
| *Os07g0567700* | SCARECROW gene regulator-like protein | 0.000120 | -3.04 |
| *Os03g0709100* | Similar to Basic blue protein | 0.000990 | 5.92 |
| *Os01g0156300* | Similar to Cappuccino protein | 0.000206 | 5.52 |
| *Os03g0711100* | Similar to CONSTANS-like protein | 0.000179 | -6.15 |
| *Os06g0654900* | Similar to CONSTANS-like protein CO6 | 0.000055 | -3.10 |
| *Os06g0141700* | Similar to Early nodulin | 0.000038 | 3.57 |
| *Os02g0264700* | Similar to Flagelliform silk protein-like protein | 0.003190 | -3.10 |
| *Os01g0546100* | Similar to Nodulin-like protein | 0.007230 | -5.33 |
| *Os05g0158600* | Similar to OsGA2ox1 | 0.003046 | -3.18 |
| *Os03g0800200* | Similar to Protein argonaute MEL1 | 0.000656 | 9.61 |
| *Os12g0117400* | Similar to RPT2-like protein | 0.000921 | -5.24 |
| *Os07g0605800* | Similar to STF-1 | 0.000774 | 9.14 |
| *Os01g0975900* | Similar to Tonoplast membrane integral protein ZmTIP1-2 | 0.009175 | -3.82 |
| *Os07g0601600* | Similar to VEP1 (VEIN PATTERNING 1); binding / catalytic | 0.000538 | -3.34 |
| *Os04g0604900* | Similar to Xyloglucan endotransglycosylase | 0.001614 | -3.68 |
| *Os06g0335900* | Similar to Xyloglucan endotransglycosylase XET2 | 0.000130 | -5.18 |
| *Os08g0538600* | Stress up-regulated Nod 19 family protein | 0.000017 | 4.11 |
| **Metabolism** |  |  |  |
| *Os01g0934800* | Alpha/beta hydrolase fold-1 domain containing protein | 0.000004 | 92.14 |
| *Os02g0705100* | Alpha/beta hydrolase fold-1 domain containing protein | 0.000578 | 3.51 |
| *Os01g0934700* | Alpha/beta hydrolase fold-1 domain containing protein | 0.003534 | 3.05 |
| *Os01g0155000* | Alpha/beta hydrolase fold-3 domain containing protein | 0.001146 | 8.31 |
| *Os11g0239500* | Alpha/beta hydrolase fold-3 domain containing protein | 0.000060 | -3.22 |
| *Os12g0135800* | Alpha/beta hydrolase fold-3 domain containing protein | 0.001092 | -3.43 |
| *Os07g0162600* | Alpha/beta hydrolase fold-3 domain containing protein | 0.000923 | -3.46 |
| *Os07g0162700* | Alpha/beta hydrolase fold-3 domain containing protein | 0.002576 | -4.04 |
| *Os07g0643000* | Alpha/beta hydrolase fold-3 domain containing protein | 0.003450 | -5.09 |
| *Os07g0643400* | Alpha/beta hydrolase fold-3 domain containing protein | 0.000311 | -6.03 |
| *Os02g0706500* | ATPase, AAA-type, core domain containing protein | 0.000744 | 4.27 |
| *Os02g0740300* | ATPase, AAA-type, core domain containing protein | 0.000214 | 3.83 |
| *Os02g0697600* | ATPase, AAA-type, core domain containing protein | 0.000631 | 3.51 |
| *Os12g0431100* | ATPase, AAA-type, core domain containing protein | 0.000905 | -10.71 |
| *Os09g0395600* | Concanavalin A-like lectin/glucanase domain containing protein | 0.000055 | -3.66 |
| *Os01g0804900* | Cytochrome P450 | 0.001229 | -3.03 |
| *Os03g0134500* | Cytochrome p450 (CYP78A9) | 0.009075 | -3.00 |
| *Os12g0134900* | Cytochrome P450 family protein | 0.000060 | 142.19 |
| *Os11g0138300* | Cytochrome P450 family protein | 0.000072 | 91.05 |
| *Os01g0803800* | Cytochrome P450 family protein | 0.000094 | 22.41 |
| *Os01g0627500* | Cytochrome P450 family protein | 0.000006 | 9.79 |
| *Os01g0803900* | Cytochrome P450 family protein | 0.000072 | 4.85 |
| *Os09g0447500* | Cytochrome P450 family protein | 0.000144 | 4.72 |
| *Os03g0760200* | Cytochrome P450 family protein | 0.002895 | 4.64 |
| *Os01g0183600* | Cytochrome P450 family protein | 0.000104 | -3.30 |
| *Os01g0211200* | Cytochrome P450 family protein | 0.000276 | -3.76 |
| *Os01g0211600* | Cytochrome P450 family protein | 0.001043 | -3.85 |
| *Os09g0447300* | Cytochrome P450 family protein | 0.001144 | -3.86 |
| *Os08g0152400* | Cytochrome P450 family protein | 0.008689 | -4.21 |
| *Os02g0124600* | D-arabinono-1,4-lactone oxidase domain containing protein | 0.003564 | -3.53 |
| *Os08g0114300* | D-arabinono-1,4-lactone oxidase domain containing protein | 0.008706 | -3.77 |
| *Os05g0429500* | Dienelactone hydrolase domain containing protein | 0.000043 | -4.17 |
| *Os06g0271500* | En/Spm-like transposon proteins family protein | 0.000009 | -4.91 |
| *Os08g0530300* | Exo70 exocyst complex subunit family protein | 0.000096 | -3.47 |
| *Os06g0548200* | FAD linked oxidase, N-terminal domain containing protein | 0.000047 | -8.95 |
| *Os03g0153400* | Fe-S metabolism associated SufE family protein | 0.000575 | -3.83 |
| *Os03g0305800* | Galactosyl transferase family protein | 0.001443 | -3.50 |
| *Os03g0306100* | Galactosyl transferase family protein | 0.000295 | -4.95 |
| *Os07g0664600* | Glucose/ribitol dehydrogenase family protein | 0.000767 | -5.27 |
| *Os08g0126300* | Glyceraldehyde-3-phosphate dehydrogenase | 0.000231 | 4.50 |
| *Os01g0956200* | Glycosyltransferase AER61, uncharacterized domain containing protein | 0.000829 | -3.32 |
| *Os01g0119000* | Glycosyltransferase AER61, uncharacterized domain containing protein | 0.006222 | -5.15 |
| *Os10g0568900* | Haloacid dehalogenase-like hydrolase domain containing protein | 0.000045 | 4.15 |
| *Os04g0587500* | HAT dimerisation domain containing protein | 0.001636 | -4.21 |
| *Os07g0232900* | Heavy metal translocating P-type ATPase family protein | 0.001069 | -3.45 |
| *Os03g0618300* | Isopenicillin N synthase family protein | 0.000713 | 5.59 |
| *Os01g0536400* | Isopenicillin N synthase family protein | 0.000068 | -3.62 |
| *Os03g0730000* | Lecithin:cholesterol acyltransferase family protein | 0.000703 | -4.95 |
| *Os12g0576600* | Metallophosphoesterase domain containing protein | 0.007940 | 3.09 |
| *Os07g0689300* | Mitochondrial ATP-dependent protease Lon precursor | 0.000317 | 3.21 |
| *Os11g0126800* | Nucleoside phosphatase GDA1/CD39 family protein | 0.009236 | 5.92 |
| *Os03g0734000* | Pentatricopeptide repeat containing protein | 0.000127 | 6.27 |
| *Os05g0557100* | Peptidase A1 domain containing protein | 0.004643 | -3.46 |
| *Os07g0532800* | Peptidase A1 domain containing protein | 0.000002 | -3.80 |
| *Os06g0610800* | Peptidase A1 domain containing protein | 0.003373 | -5.05 |
| *Os09g0473400* | Peptidase C12, ubiquitin carboxyl-terminal hydrolase 1 domain containing protein | 0.000379 | -3.30 |
| *Os10g0557900* | Peptidase M10A and M12B, matrixin and adamalysin family protein | 0.004343 | -3.65 |
| *Os03g0386800* | Peptidase S10, serine carboxypeptidase family protein | 0.000048 | 4.30 |
| *Os01g0383900* | Peptidase S59, nucleoporin family protein | 0.000171 | 5.39 |
| *Os01g0776500* | Peptidase, trypsin-like serine and cysteine domain containing protein | 0.000224 | 3.11 |
| *Os12g0263200* | Pinoresinol-lariciresinol reductase TH2 | 0.005038 | -3.04 |
| *Os10g0552200* | Plant lipid transfer protein and hydrophobic protein, helical domain containing protein | 0.002344 | -6.62 |
| *Os03g0843900* | Poly(ADP-ribose) glycohydrolase domain containing protein | 0.000149 | -6.55 |
| *Os12g0560500* | Pseudouridine synthase domain containing protein | 0.003818 | -6.16 |
| *Os08g0545000* | Pyridoxal phosphate-dependent transferase | 0.007580 | -3.50 |
| *Os08g0200100* | Ribonucleotide reductase-related domain containing protein | 0.000809 | -3.32 |
| *Os02g0707900* | Rossmann-like alpha/beta/alpha sandwich fold domain containing | 0.002131 | 3.11 |
| *Os11g0256900* | SAM dependent carboxyl methyltransferase family protein | 0.001109 | -7.03 |
| *Os06g0300000* | Short-chain dehydrogenase/reductase SDR family protein | 0.000172 | -3.39 |
| *Os04g0288100* | Similar to Adenosine diphosphate glucose pyrophosphatase precursor | 0.001640 | -4.51 |
| *Os09g0451000* | Similar to 1-aminocyclopropane-1-carboxylate oxidase 1 | 0.002711 | 3.11 |
| *Os05g0482700* | Similar to 2,3-bisphosphoglycerate-independent phosphoglycerate mutase | 0.000399 | 3.17 |
| *Os02g0713900* | Similar to 3-hydroxy-3-methylglutaryl coenzyme A reductase | 0.000012 | 6.36 |
| *Os03g0205800* | Similar to Acetyltransferase, GNAT family protein, expressed | 0.003501 | -3.42 |
| *Os01g0191200* | Similar to Acid phosphatase | 0.005097 | 4.92 |
| *Os03g0130100* | Similar to Acyl-activating enzyme 11 | 0.002194 | -4.46 |
| *Os03g0351300* | Similar to Beta-amylase | 0.003185 | -3.83 |
| *Os10g0465700* | Similar to Beta-amylase PCT-BMYI | 0.000513 | -3.05 |
| *Os04g0674800* | Similar to CEL1 | 0.000384 | -10.92 |
| *Os04g0167800* | Similar to Chalcone reductase homologue | 0.000007 | 4.17 |
| *Os04g0480600* | Similar to Cytochrome P450 71A1 | 0.000383 | 3.49 |
| *Os06g0641500* | Similar to Cytochrome P450 CYP71Y10 | 0.000913 | -4.36 |
| *Os01g0627600* | Similar to Cytochrome P450 monooxygenase CYP72A5 | 0.003018 | 11.36 |
| *Os06g0110000* | Similar to DWARF3 | 0.001921 | -4.54 |
| *Os09g0315100* | Similar to Endoribonuclease Dicer homolog 2b | 0.000027 | 3.05 |
| *Os06g0136600* | Similar to Enolase 1 | 0.000000 | 56.18 |
| *Os05g0438600* | Similar to Fructose-1,6-bisphosphatase 2 | 0.000479 | -3.36 |
| *Os09g0425200* | Similar to Glycine-rich protein | 0.002411 | 6.59 |
| *Os06g0212300* | Similar to Glycosyltransferase family-37 | 0.000300 | -4.48 |
| *Os03g0836800* | Similar to IAA-amino acid hydrolase 1 | 0.004139 | -12.61 |
| *Os03g0283200* | Similar to IN2-1 protein | 0.000027 | 6.57 |
| *Os03g0283100* | Similar to IN2-1 protein | 0.001864 | 3.47 |
| *Os09g0436500* | Similar to Ketol-acid reductoisomerase, chloroplast precursor | 0.000399 | 3.66 |
| *Os03g0273200* | Similar to Laccase | 0.002534 | 8.83 |
| *Os01g0850700* | Similar to Laccase-7 | 0.000001 | -3.17 |
| *Os03g0232800* | Similar to Lecithin:cholesterol acyltransferase family protein, expressed | 0.002984 | 6.66 |
| *Os10g0522000* | Similar to Methyltransferase family protein, expressed | 0.000447 | -3.05 |
| *Os05g0588900* | Similar to Mitochondrial chaperone BCS1 | 0.004425 | 3.52 |
| *Os04g0581300* | Similar to Mitochondrial import inner membrane translocase subunit TIM13 | 0.000268 | 3.07 |
| *Os02g0771500* | Similar to Pap1p; poly A polymerase | 0.002766 | -5.91 |
| *Os03g0417700* | Similar to Phytochrome P450 | 0.002557 | -5.19 |
| *Os09g0325700* | Similar to Protein phpsphatase 2C | 0.000028 | -5.29 |
| *Os05g0469600* | Similar to Pyruvate decarboxylase | 0.000943 | 4.28 |
| *Os01g0160100* | Similar to Pyruvate decarboxylase isozyme 2 | 0.001991 | 6.47 |
| *Os05g0307400* | Similar to Regulatory associated protein of mTOR | 0.000288 | 3.22 |
| *Os04g0229100* | Similar to Sinapyl alcohol dehydrogenase | 0.003199 | -3.71 |
| *Os05g0191700* | Similar to Stem 28 kDa glycoprotein | 0.001137 | -3.12 |
| *Os07g0624600* | Similar to Trehalose-6-phosphate phosphatase | 0.000182 | 3.42 |
| *Os01g0841600* | Similar to Triosephosphate isomerase, cytosolic | 0.000098 | 5.55 |
| *Os07g0616800* | Sucrose synthase 3 | 0.009985 | 4.11 |
| *Os12g0270900* | Sulfotransferase family protein | 0.000737 | 6.17 |
| *Os04g0340300* | Terpene synthase-like domain containing protein | 0.004911 | -10.77 |
| *Os04g0674100* | Tetratricopeptide-like helical domain containing protein | 0.003881 | -4.58 |
| *Os08g0543400* | Transferase family protein | 0.005882 | 3.14 |
| *Os06g0595800* | Transferase family protein | 0.004698 | -3.01 |
| *Os03g0185700* | Transferase family protein | 0.000436 | -4.45 |
| *Os07g0614600* | Transmembrane receptor, eukaryota domain containing protein | 0.000939 | -4.01 |
| *Os09g0442400* | t-snare domain containing protein | 0.000569 | 10.15 |
| *Os01g0597800* | UDP-glucuronosyl/UDP-glucosyltransferase family protein | 0.000137 | 22.16 |
| *Os09g0518200* | UDP-glucuronosyl/UDP-glucosyltransferase family protein | 0.005168 | 13.41 |
| *Os01g0734800* | UDP-glucuronosyl/UDP-glucosyltransferase family protein | 0.000143 | 10.81 |
| *Os03g0841600* | UDP-glucuronosyl/UDP-glucosyltransferase family protein | 0.000820 | 9.58 |
| *Os09g0517900* | UDP-glucuronosyl/UDP-glucosyltransferase family protein | 0.005375 | -3.11 |
| *Os07g0510500* | UDP-glucuronosyl/UDP-glucosyltransferase family protein | 0.001806 | -3.16 |
| *Os08g0404000* | UDP-glucuronosyl/UDP-glucosyltransferase family protein | 0.002345 | -3.25 |
| *Os07g0241800* | UDP-glucuronosyl/UDP-glucosyltransferase family protein | 0.000784 | -3.35 |
| *Os11g0457300* | UDP-glucuronosyl/UDP-glucosyltransferase family protein | 0.003269 | -4.03 |
| *Os06g0220500* | UDP-glucuronosyl/UDP-glucosyltransferase family protein | 0.002144 | -4.38 |
| **Chromatin regulation and cytoskeleton reorganization** | |  |  |
| *Os01g0189100* | Ankyrin domain containing protein | 0.000163 | 3.26 |
| *Os08g0156900* | DNA topoisomerase, type II family protein. | 0.003879 | -3.28 |
| *Os01g0580400* | Histone-fold domain containing protein | 0.001727 | -4.31 |
| *Os04g0482300* | Kelch related domain containing protein | 0.000092 | -3.04 |
| *Os08g0531100* | MAP65/ASE1 family protein. | 0.000190 | 5.01 |
| *Os02g0214900* | Oryza sativa histone deacetylase3 | 0.000011 | 12.87 |
| *Os06g0508800* | Similar to ATPase involved in chromosome partitioning | 0.007639 | -4.88 |
| *Os02g0215200* | Similar to Histone deacetylase HDAC2 | 0.003232 | 3.23 |
| *Os02g0580500* | Similar to Profilin A | 0.000892 | 6.73 |
| *Os10g0323600* | Similar to Profilin A | 0.000161 | -8.89 |
| *Os03g0841700* | Similar to Prohibitin | 0.002153 | 7.37 |
| *Os04g0462900* | Similar to Prohibitin 2 | 0.000695 | 3.52 |
| *Os02g0621100* | Similar to Su(VAR)3-9-related protein 4 | 0.000205 | 4.04 |
| **Protein with binding function or cofactor requirement** | |  |  |
| *Os03g0831400* | Beta tubulin, autoregulation binding site domain containing protein | 0.000899 | -4.22 |
| *Os06g0681200* | Cupredoxin domain containing protein | 0.002808 | 3.68 |
| *Os04g0629200* | Cupredoxin domain containing protein | 0.000010 | -3.49 |
| *Os05g0585700* | Helix-turn-helix, AraC type, subdomain 2 domain containing protein | 0.000002 | 9.24 |
| *Os09g0498500* | NAD-binding site containing protein. | 0.000550 | -5.01 |
| *Os08g0130000* | Putative DNA binding domain containing protein | 0.003300 | -3.92 |
| *Os02g0792800* | Rieske iron-sulfur protein Tic55 precursor. | 0.000400 | -5.83 |
| *Os04g0674700* | Similar to AMP-binding protein | 0.000051 | -6.08 |
| *Os07g0188000* | Similar to Argonaute 4 protein | 0.000741 | 3.72 |
| *Os01g0843300* | Similar to ATP/GTP/Ca++ binding protein | 0.000723 | 5.94 |
| *Os07g0542900* | Similar to Blue copper protein | 0.000755 | -6.31 |
| *Os06g0169900* | Similar to GOS9 protein | 0.000358 | -4.26 |
| *Os03g0805600* | Similar to Pheophorbide a oxygenase | 0.000024 | -6.53 |
| *Os12g0502100* | Similar to SKIP interacting protein 15 | 0.000001 | -8.66 |
| *Os06g0136700* | Steroid nuclear receptor, ligand-binding domain containing protein | 0.000005 | -4.10 |
| *Os08g0180500* | Zinc finger, BED-type predicted domain containing protein | 0.000206 | -5.66 |
| **Unknown** |  |  |  |
| *Os09g0240600* | (No Hit) | 0.000275 | 24.27 |
| *Os01g0803600* | (No Hit) | 0.000034 | 16.48 |
| *Os03g0716400* | (No Hit) | 0.000104 | 11.30 |
| *Os01g0322700* | (No Hit) | 0.001021 | 10.06 |
| *Os09g0263700* | (No Hit) | 0.000055 | 5.47 |
| *Os09g0500800* | (No Hit) | 0.003067 | 5.45 |
| *Os05g0376700* | (No Hit) | 0.004299 | 5.25 |
| *Os12g0523000* | (No Hit) | 0.009466 | 5.23 |
| *Os02g0163800* | (No Hit) | 0.006116 | 5.17 |
| *Os12g0564500* | (No Hit) | 0.002280 | 5.15 |
| *Os12g0288500* | (No Hit) | 0.004005 | 5.13 |
| *Os08g0209000* | (No Hit) | 0.000759 | 5.06 |
| *Os05g0376100* | (No Hit) | 0.001482 | 4.85 |
| *Os02g0773400* | (No Hit) | 0.000015 | 4.11 |
| *Os08g0384400* | (No Hit) | 0.000488 | 3.83 |
| *Os08g0177400* | (No Hit) | 0.005232 | 3.60 |
| *Os01g0762600* | (No Hit) | 0.002793 | 3.43 |
| *Os09g0330700* | (No Hit) | 0.001078 | 3.42 |
| *Os01g0791500* | (No Hit) | 0.000496 | 3.36 |
| *Os10g0108100* | (No Hit) | 0.000466 | 3.26 |
| *Os11g0668200* | (No Hit) | 0.009502 | 3.06 |
| *Os02g0585500* | (No Hit) | 0.003901 | -3.02 |
| *Os02g0614700* | (No Hit) | 0.000331 | -3.11 |
| *Os09g0440600* | (No Hit) | 0.001198 | -3.31 |
| *Os03g0133600* | (No Hit) | 0.000362 | -3.32 |
| *Os03g0734600* | (No Hit) | 0.004848 | -3.36 |
| *Os10g0485900* | (No Hit) | 0.000113 | -3.44 |
| *Os11g0488100* | (No Hit) | 0.000726 | -3.51 |
| *Os11g0249400* | (No Hit) | 0.000091 | -3.52 |
| *Os09g0496100* | (No Hit) | 0.004558 | -3.59 |
| *Os07g0561400* | (No Hit) | 0.005815 | -3.84 |
| *Os04g0562500* | (No Hit) | 0.000504 | -4.00 |
| *Os01g0564000* | (No Hit) | 0.001187 | -4.10 |
| *Os07g0527900* | (No Hit) | 0.004603 | -4.22 |
| *Os03g0580300* | (No Hit) | 0.001761 | -4.33 |
| *Os05g0108700* | (No Hit) | 0.001106 | -4.68 |
| *Os04g0308600* | (No Hit) | 0.000911 | -5.13 |
| *Os06g0238700* | (No Hit) | 0.000054 | -5.24 |
| *Os11g0260000* | (No Hit) | 0.007675 | -5.80 |
| *Os07g0600100* | (No Hit) | 0.000051 | -5.89 |
| *Os10g0333700* | (No Hit) | 0.000397 | -15.12 |
| *Os08g0155900* | Conserved hypothetical protein | 0.000005 | 83.58 |
| *Os04g0127800* | Conserved hypothetical protein | 0.000472 | 29.62 |
| *Os03g0724500* | Conserved hypothetical protein | 0.000004 | 26.74 |
| *Os07g0555100* | Conserved hypothetical protein | 0.000170 | 17.86 |
| *Os05g0147800* | Conserved hypothetical protein | 0.000031 | 15.36 |
| *Os10g0344000* | Conserved hypothetical protein | 0.002042 | 13.53 |
| *Os03g0734100* | Conserved hypothetical protein | 0.000031 | 13.10 |
| *Os11g0513900* | Conserved hypothetical protein | 0.003380 | 12.69 |
| *Os01g0510200* | Conserved hypothetical protein | 0.000064 | 8.48 |
| *Os06g0332600* | Conserved hypothetical protein | 0.000018 | 7.43 |
| *Os02g0163300* | Conserved hypothetical protein | 0.003618 | 6.54 |
| *Os05g0467600* | Conserved hypothetical protein | 0.003761 | 6.11 |
| *Os06g0239700* | Conserved hypothetical protein | 0.006801 | 6.04 |
| *Os02g0318200* | Conserved hypothetical protein | 0.000001 | 5.99 |
| *Os06g0523300* | Conserved hypothetical protein | 0.000001 | 5.32 |
| *Os07g0137600* | Conserved hypothetical protein | 0.003969 | 5.12 |
| *Os10g0509600* | Conserved hypothetical protein | 0.003592 | 4.95 |
| *Os06g0146800* | Conserved hypothetical protein | 0.002594 | 4.82 |
| *Os02g0533800* | Conserved hypothetical protein | 0.000538 | 4.30 |
| *Os01g0214500* | Conserved hypothetical protein | 0.000086 | 4.28 |
| *Os01g0206200* | Conserved hypothetical protein | 0.001284 | 3.99 |
| *Os06g0693300* | Conserved hypothetical protein | 0.001905 | 3.94 |
| *Os10g0202900* | Conserved hypothetical protein | 0.001413 | 3.66 |
| *Os08g0441400* | Conserved hypothetical protein | 0.000445 | 3.61 |
| *Os04g0672900* | Conserved hypothetical protein | 0.009683 | 3.56 |
| *Os07g0151800* | Conserved hypothetical protein | 0.000008 | 3.46 |
| *Os01g0781600* | Conserved hypothetical protein | 0.000921 | 3.41 |
| *Os10g0463900* | Conserved hypothetical protein | 0.000314 | 3.13 |
| *Os11g0678200* | Conserved hypothetical protein | 0.005546 | 3.13 |
| *Os03g0285200* | Conserved hypothetical protein | 0.000673 | 3.11 |
| *Os04g0649500* | Conserved hypothetical protein | 0.001445 | -3.00 |
| *Os08g0520700* | Conserved hypothetical protein | 0.003225 | -3.01 |
| *Os05g0500400* | Conserved hypothetical protein | 0.000112 | -3.03 |
| *Os02g0193900* | Conserved hypothetical protein | 0.002252 | -3.04 |
| *Os04g0607400* | Conserved hypothetical protein | 0.000991 | -3.04 |
| *Os03g0185800* | Conserved hypothetical protein | 0.005707 | -3.05 |
| *Os06g0218300* | Conserved hypothetical protein | 0.000296 | -3.05 |
| *Os03g0183200* | Conserved hypothetical protein | 0.000269 | -3.09 |
| *Os02g0577900* | Conserved hypothetical protein | 0.004586 | -3.10 |
| *Os02g0582800* | Conserved hypothetical protein | 0.002737 | -3.20 |
| *Os07g0109700* | Conserved hypothetical protein | 0.002653 | -3.21 |
| *Os01g0875000* | Conserved hypothetical protein | 0.003483 | -3.22 |
| *Os08g0391700* | Conserved hypothetical protein | 0.002328 | -3.22 |
| *Os06g0328400* | Conserved hypothetical protein | 0.000426 | -3.23 |
| *Os03g0204300* | Conserved hypothetical protein | 0.002369 | -3.24 |
| *Os01g0239300* | Conserved hypothetical protein | 0.001632 | -3.26 |
| *Os01g0727600* | Conserved hypothetical protein | 0.009564 | -3.27 |
| *Os01g0198500* | Conserved hypothetical protein | 0.000192 | -3.29 |
| *Os03g0349600* | Conserved hypothetical protein | 0.000327 | -3.31 |
| *Os02g0575700* | Conserved hypothetical protein | 0.006574 | -3.31 |
| *Os02g0108800* | Conserved hypothetical protein | 0.000612 | -3.41 |
| *Os03g0628800* | Conserved hypothetical protein | 0.000054 | -3.42 |
| *Os02g0828400* | Conserved hypothetical protein | 0.000709 | -3.53 |
| *Os11g0488400* | Conserved hypothetical protein | 0.004256 | -3.53 |
| *Os05g0119100* | Conserved hypothetical protein | 0.005445 | -3.58 |
| *Os01g0135600* | Conserved hypothetical protein | 0.000714 | -3.58 |
| *Os10g0100900* | Conserved hypothetical protein | 0.000171 | -3.61 |
| *Os07g0543700* | Conserved hypothetical protein | 0.003013 | -3.62 |
| *Os01g0248300* | Conserved hypothetical protein | 0.007579 | -3.68 |
| *Os07g0226200* | Conserved hypothetical protein | 0.001183 | -3.75 |
| *Os10g0159300* | Conserved hypothetical protein | 0.009070 | -3.86 |
| *Os01g0198900* | Conserved hypothetical protein | 0.009377 | -3.88 |
| *Os07g0248800* | Conserved hypothetical protein | 0.004972 | -3.97 |
| *Os03g0285300* | Conserved hypothetical protein | 0.000078 | -4.21 |
| *Os05g0479900* | Conserved hypothetical protein | 0.003126 | -4.32 |
| *Os07g0582800* | Conserved hypothetical protein | 0.001923 | -4.64 |
| *Os05g0431000* | Conserved hypothetical protein | 0.000021 | -4.64 |
| *Os01g0264600* | Conserved hypothetical protein | 0.005680 | -4.68 |
| *Os02g0438700* | Conserved hypothetical protein | 0.001478 | -4.73 |
| *Os02g0170200* | Conserved hypothetical protein | 0.000005 | -5.02 |
| *Os09g0409100* | Conserved hypothetical protein | 0.009860 | -5.04 |
| *Os06g0550800* | Conserved hypothetical protein | 0.000048 | -5.14 |
| *Os03g0776400* | Conserved hypothetical protein | 0.000436 | -5.30 |
| *Os06g0591200* | Conserved hypothetical protein | 0.002706 | -5.36 |
| *Os04g0404400* | Conserved hypothetical protein | 0.000247 | -5.50 |
| *Os05g0392900* | Conserved hypothetical protein | 0.000143 | -5.60 |
| *Os03g0826800* | Conserved hypothetical protein | 0.005817 | -5.66 |
| *Os02g0223300* | Conserved hypothetical protein | 0.000032 | -6.13 |
| *Os09g0246500* | Conserved hypothetical protein | 0.001231 | -6.37 |
| *Os10g0488400* | Conserved hypothetical protein | 0.000357 | -7.41 |
| *Os05g0372900* | Conserved hypothetical protein | 0.000564 | -8.00 |
| *Os08g0352100* | Conserved hypothetical protein | 0.000240 | -28.51 |
| *Os10g0335000* | Conserved hypothetical protein | 0.000181 | -32.11 |
| *Os05g0212400* | Conserved hypothetical protein | 0.009898 | 3.57 |
| *Os11g0513000* | Hypothetical conserved gene | 0.003558 | 9.02 |
| *Os01g0674400* | Hypothetical conserved gene | 0.002777 | 7.29 |
| *Os04g0359700* | Hypothetical conserved gene | 0.000010 | 6.89 |
| *Os12g0141600* | Hypothetical conserved gene | 0.001074 | 5.79 |
| *Os08g0239900* | Hypothetical conserved gene | 0.000101 | 4.94 |
| *Os07g0158400* | Hypothetical conserved gene | 0.000010 | 3.39 |
| *Os03g0405900* | Hypothetical conserved gene | 0.000005 | 3.15 |
| *Os02g0586900* | Hypothetical conserved gene | 0.000163 | 3.03 |
| *Os01g0774100* | Hypothetical conserved gene | 0.007276 | -3.07 |
| *Os08g0507400* | Hypothetical conserved gene | 0.002750 | -3.14 |
| *Os02g0781900* | Hypothetical conserved gene | 0.009557 | -3.38 |
| *Os05g0506800* | Hypothetical conserved gene | 0.001011 | -3.51 |
| *Os03g0776100* | Hypothetical conserved gene | 0.002487 | -3.51 |
| *Os08g0519100* | Hypothetical conserved gene | 0.000541 | -3.60 |
| *Os10g0536600* | Hypothetical conserved gene | 0.000308 | -3.97 |
| *Os06g0681300* | Hypothetical conserved gene | 0.000170 | -4.03 |
| *Os02g0455400* | Hypothetical conserved gene | 0.000874 | -4.36 |
| *Os03g0257600* | Hypothetical conserved gene | 0.000832 | -4.37 |
| *Os07g0605600* | Hypothetical conserved gene | 0.000415 | -5.02 |
| *Os11g0274700* | Hypothetical conserved gene | 0.005699 | -5.26 |
| *Os06g0692500* | Hypothetical conserved gene | 0.000212 | -5.56 |
| *Os12g0431700* | Hypothetical conserved gene | 0.002146 | -7.72 |
| *Os03g0151500* | Hypothetical protein | 0.000027 | 48.35 |
| *Os10g0386300* | Hypothetical protein | 0.000186 | 21.31 |
| *Os01g0694600* | Hypothetical protein | 0.000773 | 16.75 |
| *Os03g0724600* | Hypothetical protein | 0.000028 | 15.26 |
| *Os04g0401900* | Hypothetical protein | 0.000538 | 15.23 |
| *Os06g0295700* | Hypothetical protein | 0.000006 | 11.18 |
| *Os11g0532200* | Hypothetical protein | 0.000380 | 9.30 |
| *Os11g0513500* | Hypothetical protein | 0.001634 | 9.17 |
| *Os03g0734200* | Hypothetical protein | 0.000092 | 8.07 |
| *Os12g0162800* | Hypothetical protein | 0.000863 | 7.11 |
| *Os03g0129400* | Hypothetical protein | 0.006363 | 6.58 |
| *Os06g0146600* | Hypothetical protein | 0.000092 | 5.59 |
| *Os12g0111000* | Hypothetical protein | 0.005335 | 4.82 |
| *Os09g0495500* | Hypothetical protein | 0.000539 | 4.25 |
| *Os11g0516800* | Hypothetical protein | 0.000017 | 3.60 |
| *Os03g0622500* | Hypothetical protein | 0.002486 | 3.10 |
| *Os12g0573000* | Hypothetical protein | 0.002257 | -3.03 |
| *Os02g0332700* | Hypothetical protein | 0.000309 | -3.05 |
| *Os09g0553900* | Hypothetical protein | 0.000813 | -3.15 |
| *Os06g0510200* | Hypothetical protein | 0.000085 | -3.15 |
| *Os05g0500200* | Hypothetical protein | 0.001147 | -3.16 |
| *Os08g0394000* | Hypothetical protein | 0.000563 | -3.16 |
| *Os01g0888900* | Hypothetical protein | 0.003280 | -3.19 |
| *Os02g0738800* | Hypothetical protein | 0.000501 | -3.24 |
| *Os05g0406700* | Hypothetical protein | 0.000875 | -3.25 |
| *Os12g0171300* | Hypothetical protein | 0.000793 | -3.26 |
| *Os01g0392600* | Hypothetical protein | 0.009015 | -3.32 |
| *Os03g0668500* | Hypothetical protein | 0.001729 | -3.39 |
| *Os03g0575700* | Hypothetical protein | 0.001071 | -3.44 |
| *Os01g0187200* | Hypothetical protein | 0.003815 | -3.54 |
| *Os05g0142600* | Hypothetical protein | 0.000068 | -3.71 |
| *Os05g0208500* | Hypothetical protein | 0.001033 | -3.75 |
| *Os06g0661500* | Hypothetical protein | 0.000266 | -3.77 |
| *Os08g0474800* | Hypothetical protein | 0.000450 | -3.79 |
| *Os06g0673700* | Hypothetical protein | 0.005862 | -3.79 |
| *Os07g0237700* | Hypothetical protein | 0.001750 | -3.86 |
| *Os09g0524500* | Hypothetical protein | 0.000435 | -3.87 |
| *Os05g0237300* | Hypothetical protein | 0.002374 | -4.85 |
| *Os06g0708200* | Hypothetical protein | 0.000330 | -5.56 |
| *Os05g0560200* | Hypothetical protein | 0.001739 | -5.97 |
| *Os03g0608000* | Hypothetical protein | 0.000641 | -6.70 |
| *Os06g0493100* | Hypothetical protein | 0.001338 | -7.25 |
| *Os03g0643900* | Hypothetical protein | 0.001961 | -7.28 |
| *Os06g0293500* | Hypothetical protein | 0.001017 | -8.42 |
| *Os09g0115900* | Non-protein coding transcript | 0.006155 | -4.02 |
| *Os03g0836900* | Non-protein coding transcript | 0.003256 | -6.14 |
| *Os02g0628900* | Non-protein coding transcript, uncharacterized transcript | 0.002820 | 3.72 |
| *Os05g0195000* | Non-protein coding transcript, uncharacterized transcript | 0.006556 | 3.29 |
| *Os03g0582900* | Non-protein coding transcript, uncharacterized transcript | 0.001896 | -3.03 |
| *Os09g0498400* | Non-protein coding transcript, uncharacterized transcript | 0.000060 | -4.42 |
| *Os09g0246300* | Non-protein coding transcript, uncharacterized transcript | 0.004428 | -5.72 |
| *Os03g0787400* | Non-protein coding transcript, unclassifiable transcript | 0.003167 | -3.55 |
| *Os08g0139400* | Non-protein coding transcript, unclassifiable transcript | 0.001818 | -3.80 |
| *Os07g0575900* | Plant protein of unknown function DUF946 family protein | 0.000146 | -3.70 |
| *Os05g0568800* | Protein of unknown function DUF1645 family protein | 0.000512 | -3.20 |
| *Os08g0356500* | Protein of unknown function DUF247, plant family protein | 0.000067 | -4.30 |
| *Os08g0356700* | Protein of unknown function DUF247, plant family protein | 0.002426 | -4.39 |
| *Os01g0680200* | Protein of unknown function DUF250 domain containing protein | 0.000117 | -3.98 |
| *Os04g0322100* | Protein of unknown function DUF26 domain containing protein | 0.009934 | -3.18 |
| *Os04g0316200* | Protein of unknown function DUF26 domain containing protein | 0.002770 | -3.29 |
| *Os04g0659300* | Protein of unknown function DUF26 domain containing protein | 0.000013 | -12.79 |
| *Os12g0635500* | Protein of unknown function DUF266, plant domain containing protein | 0.000837 | -3.64 |
| *Os03g0120100* | Protein of unknown function DUF284, transmembrane eukaryotic family protein | 0.000125 | 3.51 |
| *Os01g0719000* | Protein of unknown function DUF581 family protein | 0.001904 | 3.12 |
| *Os05g0531100* | Protein of unknown function DUF584 family protein | 0.000048 | 3.94 |
| *Os03g0113900* | Protein of unknown function DUF584 family protein | 0.000672 | -4.94 |
| *Os04g0154800* | Protein of unknown function DUF594 family protein | 0.004987 | -3.07 |
| *Os08g0153900* | Protein of unknown function DUF599 family protein | 0.000517 | 7.63 |
| *Os03g0233000* | Protein of unknown function DUF607 family protein | 0.004159 | -4.58 |
| *Os01g0852400* | Protein of unknown function DUF740 family protein | 0.000964 | -3.21 |
| *Os04g0675000* | Protein of unknown function DUF789 family protein | 0.000361 | 4.12 |
| *Os05g0149500* | Protein of unknown function DUF821, CAP10-like domain containing | 0.000066 | -3.41 |
| *Os04g0116200* | Protein of unknown function DUF827, plant family protein | 0.000031 | 5.12 |
| *Os11g0256200* | Protein of unknown function DUF842, eukaryotic family protein | 0.000002 | 3.19 |
| *Os09g0416600* | Protein of unknown function DUF868, plant family protein | 0.001108 | -3.29 |
| *Os03g0152900* | Similar to predicted protein | 0.000041 | 4.36 |
| *Os01g0221600* | Similar to predicted protein | 0.000978 | 3.77 |
| *Os06g0301300* | Similar to predicted protein | 0.000398 | 3.38 |
| *Os01g0220500* | Similar to predicted protein | 0.000042 | 3.30 |
| *Os07g0170200* | Similar to predicted protein | 0.000107 | -3.08 |
| *Os01g0127200* | Similar to predicted protein | 0.000498 | -3.36 |
| *Os10g0566200* | Similar to predicted protein | 0.001257 | -3.41 |
| *Os05g0338900* | Similar to predicted protein | 0.007006 | -3.58 |

^a^ Name of probe set on Agilent Rice GeneChip.

^b^ Gene annotation in The Rice Annotation Project Database.

^c^ P-value of statistical Student’s *t* test.

^d^ Fold change of *alt1* compared with WT. Value is calculated by R-software.

^e^ Genes in bold indicate those involved in abiotic stress response.
