# Supplementary material for: ALT1, a Snf2 Family Chromatin Remodeling ATPase, Negatively Regulates Alkaline Tolerance through Enhanced Defense against Oxidative Stress in Rice
Source: PLoS One. 2014 Dec 4;9(12):e112515. doi: 10.1371/journal.pone.0112515 (PMC4256374; doi:10.1371/journal.pone.0112515)
Supplement: Table S3 — Primers used in this study. (DOCX) [file pone.0112515.s007.docx]

**Table S3. Primers used in this study.**

| **Primer name** | **Primer sequence (5'-3')** | | |
| --- | --- | --- | --- |
| **For map-based cloning** | |  |  |
| M1-F | CGGCTCGTCGCCTCGTAGAGAT | | |
| M1-R | GACGGCGACATGAATCATGATGCATC | | |
| M6-F | ACCGGATTGGCACGACACGC | | |
| M6-R | GATGTACTCCGTTGTCTCCTACG | | |
| **For sequencing analysis** | |  |  |
| M13-F | GTAAAACGACGGCCAGT | | |
| M13-R | CAGGAAACAGCTATGAC | | |
| S2-F | GGTGATGTGCCTGGAAGCATGC | | |
| S2-R | CTGCAGATATTCTCCTTGTACAGTTAGGC | | |
| S3-F | GTCTTGCTAGTGGACATGTGTTGCC | | |
| S4-F | GGTAGCAACCGCACAAAGGATC | | |
| S5-F | GAGCGTGCTTTCTATAACACTCTCG | | |
| 3423-F | GTACTCCCTCTGAATATGCTGGCAC | | |
| **For transgenic construction** | |  |  |
| C1-F | TCTAGAATGGAGGAAGCGGCGGCG | | |
| C1-R | GAGCTCCTAAACCATAAACAAATAGTTCAAG | | |
| P1-F | TCTAGACACTCCTTACCCTCGACGGCG | | |
| P1-R | AAGCTTATGCGCGTATCAATCCGACAAACACC | | |
| RNAi -F | TCTAGATCGACCTGTCACAGTGTCACGGT | | |
| RNAi -R | ACTAGTCTGATGAGTTGCCACTTGCACGGAG | | |
| GFP-F | TCTAGAATGGAGGAAGCGGCGGCGGC | | |
| GFP-R | AGATCTGGCAACCATAAACAAATAGTTCAAGTC | | |
| **For quantitative real-time PCR** | |  |  |
| *OsSWAP70B*-F | AATGCGACTGCAATCGAAGCATCC | | |
| *OsSWAP70B*-R | TTTGTAGTTGAGCCCAAAGCTGCC | | |
| *OsProDH2*-F | CTCCTTCGACAGACAGCTGCT | | |
| *OsProDH3*-R | TAAGGCAGCCTCAGTCTTCTGC | | |
| *Os05g0331200*-F | TGAAGCAAGTTGGTCAGGCAAACC | | |
| *Os05g0331200*-R | TTGTCCTTGTCTGCTACCCGGAAA | | |
| *Os05g0573200*-F | AGTGAAGGAGGCTATGTTTGGGCT | | |
| *Os05g0573200*-R | TGAACACGGTAATGACGGGTGACA | | |
| *Os02g0318100*-F | TCGATCTGACGAAACACCACAACC | | |
| *Os02g0318100*-R | TCCTCTGGAAGAAGAGGTCAACCG | | |
| *OsAOX1a*-F | TTCTTCAACGCCTACTTCCTGGGT | | |
| *OsAOX1a*-R | TTGAGGAACTCGGTGTACGAGTGGAT | | |
| *OsAOX1b*-F | ATCACAGCAACACAAGCCAACCAC | | |
| *OsAOX1b*-R | TGTGATGATTCTCAGAGCGCCCAA | | |
| *OsGSTU5*-F | AAGGCCAAGTTTGGCGCCAAGT | | |
| *OsGSTU5*-R | AGGTAGGAGCAAAATCACAACGCG | | |
| *OsGSTU19*-F | GTGAGGCCAGTTGAAGTTTGCTGA | | |
| *OsGSTU19*-R | AAGATACACGGTGCGGCAATACGA | | |
| *OsGSTU37*-F | TAGCAACCCTGTCCACAAGAAGGT | | |
| *OsGSTU37*-R | ATGAATTGAGCCCAGAAACGAGCC | | |
| *OsGSTU39*-F | GCCTGCTTTCGTCTTCGTGGATTT | | |
| *OsGSTU39*-R | ACGCGTGTCAACGTACTCCTCAAA | | |
| *OsGSTF5*-F | AGGGCAAATTCAGCTGCAAGAAGG | | |
| *OsGSTF5*-R | TGGAATCTCACCGAACGGGTTTCT | | |
| *OsGSTU41*-F | TGATAAGTTCTCGAGGCCGTTCTG | | |
| *OsGSTU41*-R | ATTCTCCTTCGCTTCCCTCACGAA | | |
| *OsGSTU4*-F | TCATTCTCTGTTCCGCTCTCTCAC | | |
| *OsGSTU4*-R | TCTTGTTGACGAGGTCCTGCTTGA | | |
| *OsGSTU16*-F | ATGTGATGACTAGTGATGCGGCCT | | |
| *OsGSTU16*-R | CCTTGCACAAGCACAACGAACAAC | | |
| *OsGSTU29*-F | GATGAAGTTGGTGTTGGTGTGCCA | | |
| *OsGSTU29*-R | GCAAAGTTCACACAGTCGTCACATCG | | |
| *OsREC8*-F | TCACCCAGTGGACTTTGCAGATGA | | |
| *OsREC8*-R | AGCTTGAAGCAGACCTGCGACTTA | | |
| *Os01g0939300*-F | TAGTTTGATGCCCAGTGTACCGCT | | |
| *Os01g0939300*-R | TGCTTTCTCCTTCGCCTGCTAGAT | | |
| *OsRAD51*-F | GAATGTAGCTTATGCAAGAGCA | | |
| *OsRAD51*-R | AGGCTGTTGCACTATCTACAATC | | |
| *OsDMC1A*-F | GAAGCTGTTTTCCAGGTAACATC | | |
| *OsDMC1A*-R | CCCATTTTAAGGTATAATCGTCTGG | | |
| *OsNSE4*-F | TGCTGCTAGTGCCATAACCTCAG | | |
| *OsNSE4*-R | CGTCGACGACCATGTCTTTCATGA | | |
| *Os07g0137000*-F | TTCACTCGAGCCCAAAGGTTGAGA | | |
| *Os07g0137000*-R | ACTGACGCAGAGGCGCTACATAAT | | |
| *OsbZIP25*-F | TGACAACCGAAGTCCAACGCCTAA | | |
| *OsbZIP25*-R | CATTGTTCTTCGCTGCACCGTTCT | | |
| *OsNAC085*-F | TGAAAGTAACTCCTGTGCCCACCA | | |
| *OsNAC085*-R | TTGATCTTGGTTGTCAGGAGGCCA | | |
| *OsNAC045*-F | TCGTCTCCCAAATCCTCTTGGCAT | | |
| *OsNAC045*-R | GCACGTAATTAAGCAGCAGCAGCA | | |
| *ALT1*-F | GTTAAAGGTGGCATTGTGGTGCGA | | |
| *ALT1*-R | AACCATACGGCTGAAATGCGAAGC | | |
| *Actin*-F | TGACGGAGCGTGGTTACTCATTCA | | |
| *Actin*-R | TCTTGGCAGTCTCCATTTCCTGGT | | |
|  |  | | |
